# CovET: A covariation-evolutionary trace method that identifies protein structure–function modules

**DOI:** 10.1016/j.jbc.2023.104896

**Published:** 2023-06-07

**Authors:** Daniel M. Konecki, Spencer Hamrick, Chen Wang, Melina A. Agosto, Theodore G. Wensel, Olivier Lichtarge

**Affiliations:** 1 Quantitative and Computational Biosciences Graduate Program, Baylor College of Medicine, Houston, Texas, USA; 2 Chemical, Physical, and Structural Biology Graduate Program, Baylor College of Medicine, Houston, Texas, USA; 3 Department of Molecular and Human Genetics, Baylor College of Medicine, Houston, Texas, USA; 4 Verna and Marrs McLean Department of Biochemistry and Molecular Biology, Baylor College of Medicine, Houston, Texas, USA; 5 Cancer and Cell Biology Graduate Program, Baylor College of Medicine, Houston, Texas, USA; 6 Computational and Integrative Biomedical Research Center, Baylor College of Medicine, Houston, Texas, USA

**Keywords:** protein motifs, allosteric determinants, coevolution, epistasis, functional sites

## Abstract

Measuring the relative effect that any two sequence positions have on each other may improve protein design or help better interpret coding variants. Current approaches use statistics and machine learning but rarely consider phylogenetic divergences which, as shown by Evolutionary Trace studies, provide insight into the functional impact of sequence perturbations. Here, we reframe covariation analyses in the Evolutionary Trace framework to measure the relative tolerance to perturbation of each residue pair during evolution. This approach (CovET) systematically accounts for phylogenetic divergences: at each divergence event, we penalize covariation patterns that belie evolutionary coupling. We find that while CovET approximates the performance of existing methods to predict individual structural contacts, it performs significantly better at finding structural clusters of coupled residues and ligand binding sites. For example, CovET found more functionally critical residues when we examined the RNA recognition motif and WW domains. It correlates better with large-scale epistasis screen data. In the dopamine D2 receptor, top CovET residue pairs recovered accurately the allosteric activation pathway characterized for Class A G protein-coupled receptors. These data suggest that CovET ranks highest the sequence position pairs that play critical functional roles through epistatic and allosteric interactions in evolutionarily relevant structure-function motifs. CovET complements current methods and may shed light on fundamental molecular mechanisms of protein structure and function.

Covariation analysis probes the multiple sequence alignment of a protein family to search for sequence positions whose mutational pattern of variations are not independent. Since most compensatory couplings are thought to arise from structurally neighboring residues, covariant residue positions have been sought to predict structural contacts and thus constrain the search space of possible protein folds toward *de novo* computational structure prediction (1, 2, 3, 4, 5, 6). AlphaFold (7, 8) and RoseTTAFold (9) both use covariation among many other training features in the context of machine learning.

The assumptions of structural proximity and compensatory mutations in covariation analysis bear closer scrutiny, however. First, concerted motions within a protein may lead to allosteric interactions. As a result, compensatory mutations may occur between structurally distant protein residues (10, 11, 12). Second, coupled residues must not necessarily undergo compensatory mutations during evolution. In fact, phylogenetic divergence involves changes in functional aptitude, with larger changes between increasingly distant species. Accordingly, mutational perturbations are increasingly less likely to be compensated between evolutionary distant orthologs—since mutation is precisely the instrument of evolution. These allosteric and evolutionary caveats are likely to impact the analysis of large protein sequence alignments but are not accounted for by current covariation algorithms in their search for coupled pairs of residues in structural contact (13, 14).

Therefore, we used the Evolutionary Trace (ET) framework to better account for phylogenetics in covariation analysis of structural contacts, allosteric interactions, and epistasis following a prior but different effort (15, 16). ET ranks every sequence position by its relative tolerance or sensitivity to mutation by correlating sequence variations with phylogenetic divergences between species. This approach has helped predict protein binding sites (17, 18), design separation of function mutations (19, 20, 21, 22), annotate and alter protein function (23, 24, 25, 26, 27, 28, 29, 30, 31, 32, 33, 34, 35, 36), design inhibitors (37, 38), and identify residues involved in diseases (39, 40). ET algorithms iteratively divide a multiple sequence alignment into progressively smaller sequence groupings following the branching pattern of the phylogenetic tree, penalizing each grouping by the total amount of entropy in each group. Accordingly, positions with patterns of variations that track closely with the evolutionary tree are deemed to be most functionally significant and penalized least. Positions that vary irrespective of the evolutionary tree and are deemed least important and penalized severely (17, 41). Here, we reasoned that the same ET penalty scheme could be extended to pairs of residue positions, as to measure how well their variations are consistent with phylogenetic divergences. And thus developed our new covariation algorithm, CovET, which ranks the relative tolerance of residue pairs to mutations using the ET framework.

We tested CovET on both a large scale and in specific structures for its ability to predict structural features, functional sites, allosteric interactions, and experimental data on epistasis. We find that CovET not only identifies covarying residues that correspond to individual structural contacts of interest but outperforms other methods in the ability to identify mutually clustered pairs of covarying residues that reveal functional sites and pathways linked to allostery and to epistasis. The code for CovET is open and freely available on GitHub at: https://github.com/LichtargeLab/Covariation-ET.

## Results

### CovET method development

**Figure 1:**
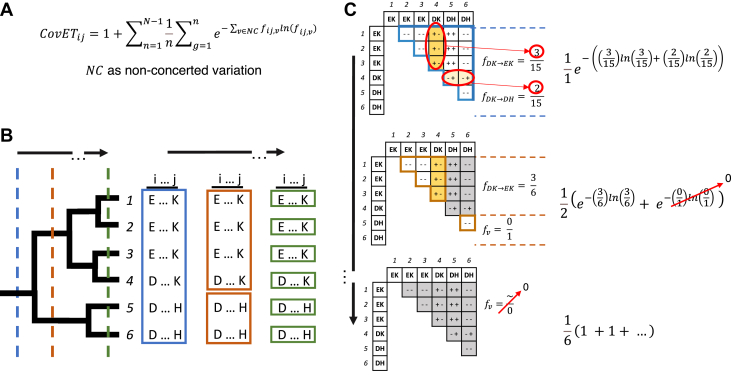
**CovET Pipeline.***A*, CovET Equation, *ij* is a pair of residues, N is the number of sequences in the alignment, n is the division in the tree, g is the group of the tree at division n, v is one of the 17,640 possible nonconcerted variations in a 21 character alphabet, *f*_*ijv*_ is the frequency of nonconcerted variation v in group g. *B*, *vertical dashed lines* show division of the tree into groups (*blue* n = 1, *orange* n = 2, *green* n = 6), boxed sequences are resulting groups (*g*) from each division. *C*, the substitution matrix for each division in the tree. Instances of nonconcerted variation are shown in different shades of *yellow* and are used in the penalty function (*right*). The boxed regions of the matrix represent the pairwise comparison, defined on a per group basis (second substitution matrix has two boxed regions, corresponding to the two groups in the tree at that division). In each box the comparison of the residues in each pair is indicated by the + and - symbols, with + meaning variation and - meaning conservation (*i.e.*, ++ concerted variation, -- conservation, and +- or -+ non-concerted variation).

CovET uses the ET framework to identify phylogenetically supported residue couplings. ET has the general form: ${ET}_{i}=\sum_{n=1}^{N}\frac{1}{n}\sum_{g=1}^{n}x_{i}$, where i is a position or pair of positions, N is the depth of the phylogenetic tree, n is a specific level in the tree, g is a group of evolutionarily related sequences (represented by a branch in the tree), and $x_{i}$ is a scoring function for residues at position i in group g in the phylogenetic tree. This general algorithm captures the process of traversing the phylogenetic tree from root to leaves (n=1 to N, represented by vertical-colored lines in Figure 1*B*) and of characterizing each group (g, represented by colored boxes in Fig. 1*B*) according to a penalty function $x_{i}$. CovET penalizes nonconcerted variations (AB > AC or AB > CB) between pairs of residues according to the exponential of the Shannon entropy, *i.e*., the diversity metric or perplexity, resulting in the final formula in Equation 2 and Figure 1. v in the entropy calculation is any of the nonconcerted variations which can be observed when comparing a pair of positions between one sequence and another, a process highlighted in Figure 1*C*. CovET ignores concerted variation (AB > CD) and conservation (AB > AB) as neither is inconsistent with a coupling. The score resulting from a simple characterized pair of positions ij is demonstrated in Figure 1*C*. We hypothesize that this phylogenetic formulation will allow us to identify functionally relevant residue couplings.

### CovET preferentially identifies higher order protein structures over local contacts

**Figure 2:**
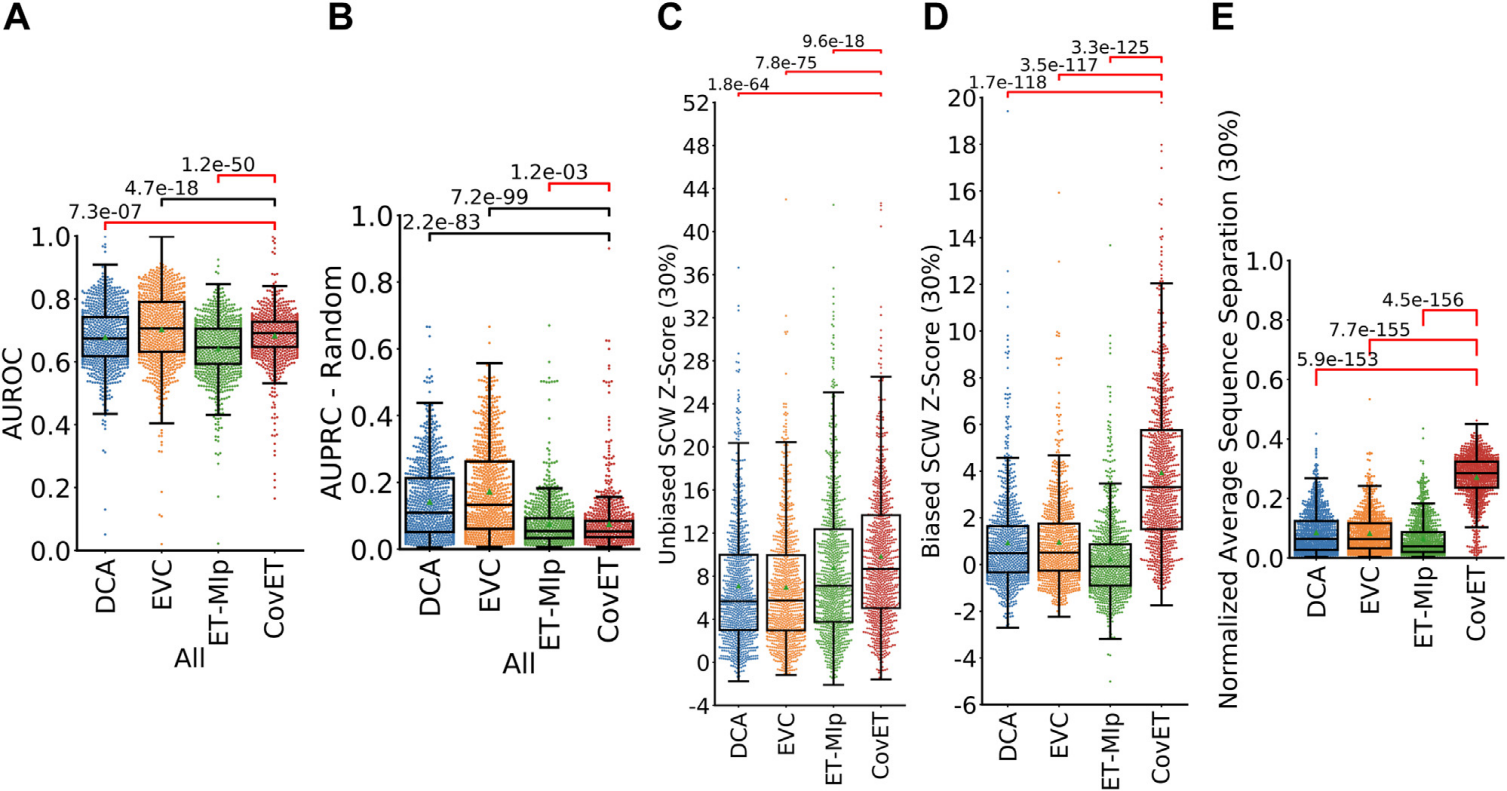
**CovET recapitulates the performance of existing methods in predicting structural contacts, but its predictions are more significantly clustered structurally.***A*, AUROC measured for all contacts (residues where Cβ are within 8 Å of each other, C⍺ for glycine) at least six residues apart. Pane (*B*) shows the same contacts measured at the same sequence separation categories evaluated by AUPRC. This evaluation which is less influenced by the class imbalance between contacts and noncontacts. The expected AUPRC value from a random predictor is the positive rate in the standard, which is the number of contacts/total residue pairs. AUPRC—Random evaluates whether the predictor performs better than random. *C* and *D*, unbiased (*C*) and biased (*D*) Selection cluster weighting (SCW) z-scores measured for each protein and each of the tested methods. While all methods show significant (z-score >2) clustering for most proteins, CovET shows significantly more clustering than DCA, EVCouplings (EVC), and ET-MIp in unbiased SCW z-scores and significantly better clustering than the three other methods in biased SCW z-scores. *E*, the average distance between pairs at 30% coverage is much higher for CovET than for any of the other methods assessed, confirming that the increase in biased SCW Z-Scores is due to clustering of residues which are significantly further apart in primary sequences. Significance was measured using the paired, two-sided Wilcoxon rank sum test. When CovET significantly outperforms other methods, the comparison bars are colored *red*. AUPRC, area under the precision recall curve.

To test whether CovET identifies local structural contacts (residues where Cβ are within 8 Å of each other, C⍺ for glycine), we studied its rankings of residue pairs in 943 protein families. ET and other alignment-based methods are inherently sensitive to the alignment itself, so we used the Pfam dataset to obtain a large, diverse protein set (42) (Table S1, see Experimental Procedures for detail). We compared CovET against standard methods: EVCouplings (13, 43) and DCA (44) as well as our previous covariation algorithm, ET-MIp which used the same ET framework but used mutual information to identify covariant residues (16). Based on the area under the receiver operating curve (AUROC, Fig. 2*A*), CovET improved over DCA and ET-MIp in predicting structural contacts, especially in the medium and long-range categories (Fig. S1) but was outperformed by EVCouplings. Based on the area under the precision recall curve (AUPRC, Fig. 2*B*), which is less sensitive to the class imbalance between positive and negative cases, CovET only outperforms ET-MIp. Importantly, all methods performed better than what would be expected from a random predictor.

A hallmark of phylogenetic strategies to predict the functional importance of individual residues is that the top predictions cluster structurally in 3D space (45, 46, 47, 48), which would be useful in higher order protein structure and function determination. Therefore, we asked next whether covarying residue pairs identified by CovET and other methods were structurally random or clustered into subregions of structural and functional importance. For this, we converted the pairwise covariation scores for each of the 943 proteins in our Pfam dataset to single residue scores (based on the order of first appearance in a pair) and applied two complementary measures of structural clustering: the biased and unbiased selection cluster weighting (SCW) z-scores (41, 45, 46). Both scores measure how clustered a group of residues are on a given protein structure, but the biased SCW z-score gives greater weight to clusters of residues more distant in sequence. SCW z-scores were measured for the top 30% of predicted residues in accordance with previous studies (45, 46). While all methods achieved significant clustering (unbiased SCW z-score >2) in most proteins (Fig. 2*C*), top-ranked CovET pairs are more significantly clustered than those of EVCouplings, DCA, and ET-MIp (CovET mean unbiased Z-Score 9.9, DCA 7.1, EVCouplings 7.0, ET-MIp 8.9). This advantage grew when measured by the biased SCW z-score (CovET mean biased Z-Score 3.9, DCA 0.9, EVCouplings 1.0, ET-MIp 0.1, Fig. 2*D*), with CovET strongly outperforming all other methods. These results showed that CovET is better at identifying amino acid structural clusters than individual local contacts, which is fundamentally different from the other three covariation methods.

Finally, given CovET’s performance in the biased SCW z-score, we compared the average sequence separation (normalized by protein length) of top predicted pairs for all methods (Fig. 2*E*). DCA, EVCouplings, and ET-MIp disproportionately rank pairs of residues that are close together in the sequence among their top results. In sharp contrast, pairs predicted by CovET are farther apart across the sequence. These data show that CovET pairs are qualitatively and structurally different. They involve more distant positions in the sequence (Fig. 2*E*), yet these cluster together in the protein structure much better than the pairs predicted by other methods. Together, these results show that CovET captures coevolutionary relationships involving sequence neighbors, like other methods, but also positions far apart in the primary sequence, a unique property of this method.

### CovET identifies important functional residues

**Figure 3:**
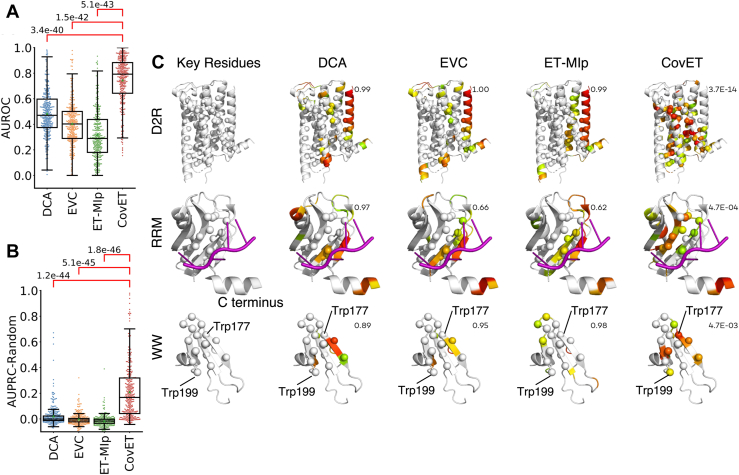
**Top CovET residues significantly overlap with known functional sites.***A* and *B*, CovET performs the best in recovering ligand binding sites in the pfam dataset judging by both AUROC (*A*) and AUPRC (*B*). Similar to Figure 2*B*, the AUPRC values are adjusted with the AUPRC of random predictors for each protein. Significance was measured using the paired, two-sided Wilcoxon rank sum test. When CovET significantly outperforms other methods, the comparison bars are colored *red*. *C*, *top* CovET predictions significantly overlap with known functional residues in the dopamine 2 receptor, RRM, and WW domain. Known functional residues visualized as spheres. Top 30% of residues shown on a *red* to *green color* scale, with *red* indicating the most highly ranked residues, and *green* those just at the 30% threshold. False positives shown as *color scaled ribbons*, false negatives shown as *white spheres*. Significance was measured using the one-sided hypergeometric test. The overlap between *top* predictions and known functional residues are evaluated with hypergeometric test. *p*-values are shown to the *upper right* of each structure. AUPRC, area under the precision recall curve; AUROC, area under the receiver operating curve; D2R, dopamine D2 receptor; RRM, RNA recognition motif.

Having confirmed the structural clustering of top CovET predictions, we assessed CovET’s ability to recover functionally important residues, like other phylogenetic methods which cluster well. It is impractical to manually gather the functionally important residues for every protein in the Pfam dataset. Instead, we turned to the BioLiP database to gather information on biologically relevant ligands in our Pfam queries (49). Defining residues within 4 Å of biologically relevant ligands as being functionally important, we could characterize them in 329 of our Pfam queries from the BioLiP database. As shown in Figure 3*A*, CovET was the best at recovering these functionally important residues with an average AUROC of 0.746, while the other three methods have average AUROCs below random (0.5). As measured by AUPRC, CovET still significantly outperforms the other methods (Fig. 3*B*). These results suggested that unlike other methods, CovET is uniquely able to identify functionally relevant residue pairs, as opposed to solely structural neighbors.

To assess CovET’s ability to identify functional sites in greater detail, we gathered key functional site information from the literature for two protein domains (the RNA recognition motif (RRM) domain and the WW domain) and for the single-domain dopamine D2 receptor (D2R) and tested how well CovET and the other covariation methods could recover their functional sites. Multiples sequence alignments of each were obtained (see Experimental Procedures, Fig. S2 and Table S2) and covariation analyses performed. The RRM domain is found across a wide variety of organisms including eukaryotes, prokaryotes, and viruses (50, 51, 52, 53). This domain is ∼90 amino acids long and plays a role in almost all stages of RNA processing (51, 53, 54, 55, 56, 57). The RRM domain is built mainly from a β-sheet that contains two highly conserved motifs, RNP1 and RNP2, that are essential to the domain’s main function, binding poly(A) RNA (50, 51, 53, 55, 58). CovET picked out many of the key residues from the β-sheets that bind RNA and others in the C-terminal region that stabilizes the RNA binding network. CovET significantly recovers the RNP1 motif at low coverage, with *p*-values of 0.042 at 20% coverage and 0.008 at 30%, while the other methods failed to identify this site by 30% coverage (Figs. 3*C* and S3; Table S3). At 30% coverage, DCA, EVCouplings, and ET-MIp identified some residues in the C-terminal region, but these were disconnected from the few residues these methods recovered in the β-sheets (Fig. S4). In sharp contrast, the top pairs predicted by CovET connected the RNA binding β-sheets and the C terminus. These data show that CovET preferentially identifies key sequence positions in the RRM domain. Additionally, top CovET predictions formed a dense and interconnected network in the RRM domain: many of the top pairs had overlapping residues. In contrast, top pairs from other methods form a sparser network and reach a given residue coverage with fewer pairs than CovET. For the RRM and WW domains as well as the D2R, CovET has more residues pairs and a denser network for a given coverage cutoff (Figs. S4–S6). This is because coverage cutoffs are defined as all the pairs it takes to recover a certain percent of residues in the protein. For example, a 10% cutoff is defined as all the residue pairs it takes until 10% of residues are recovered by at least one pair. Methods whose top pairs repeatedly select the same residues will have more pairs for a given coverage. CovET consistently takes more pairs to reach a certain coverage, which highlights the interconnectedness of top CovET pairs.

We next assessed the recovery of key residues in the WW domain. WW domains are 30 to 50 amino acids long and have one of the smallest spontaneously folding β-sheet structures, consisting of three anti-parallel β-strands (59, 60, 61, 62, 63, 64, 65, 66). This domain enables protein–protein interactions by binding short peptide sequences (*e.g*., PPxY in hYAP65) (59, 60, 61, 62, 63, 64, 65, 66). The most important sites include the two tryptophans (W) for which the WW domain is named (59, 60, 61, 65, 66) and two hydrophobic patches, one involved in binding and the other in stability (61, 63, 64, 65). At 10% coverage, CovET recovers one titular tryptophan, while the second one is recovered at 20% coverage. Neither is recovered by DCA, EVCouplings, or ET-MIp by 30% coverage (Fig. 3*C* and Table S4). At 30% coverage, CovET is the only method to significantly recover the combined set of conserved residues and functional sites (Figs. 3*C* and S5). None of the other covariation methods reaches significance for the highlighted sites or the combined set of conserved residues and functional sites. In addition, the WW domain contains residues that can be mutated without causing significant perturbation to its structure or function, dubbed insensitive residues here, mostly in the N and C termini and the turns between β-strands (61, 65). Here, the pattern is reversed, and CovET does not pick up any insensitive residues by 30% coverage, while EVCouplings and ET-MIp begins to pick these residues up at 10% coverage and DCA by 20% coverage (Table S5). These data show that CovET preferentially identifies key conserved residues and functional sites in the WW domain, while DCA, EVCouplings, and ET-MIp recover known variable positions.

### CovET highlights functional networks in the D2R

If top-ranked CovET residue pairs have important structural and functional interactions, we would expect that they would form highly interconnected networks in proteins that mediate long-range information transfer across a protein *via* allosteric pathways. To test this hypothesis, we focused on the D2R, a member of the Class A G protein-coupled receptor (GPCR) family, the single largest family of eukaryotic proteins (67, 68). Class A GPCR’s have arguably the most-studied allosteric signaling mechanism that depends on the concerted motions of its seven transmembrane helices, in which various functional modules have been described in detail (36, 67, 68, 69, 70, 71, 72, 73, 74, 75, 76, 77).

**Figure 4:**
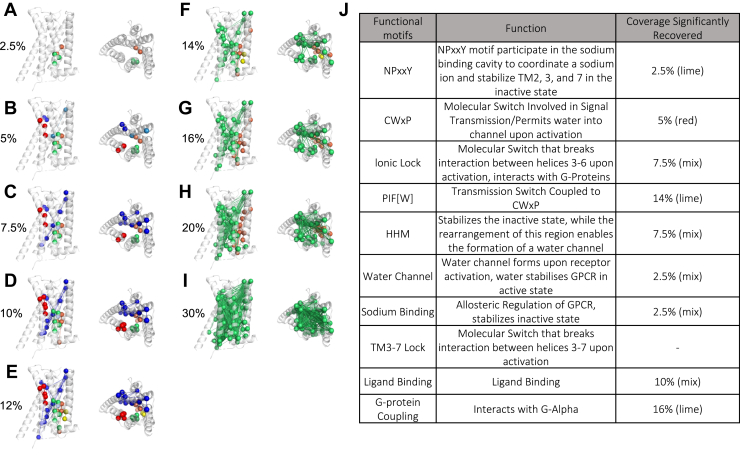
**CovET predictions highlight functional network in the dopamine D2 receptor.***Top* CovET pairs at different coverages are displayed as *lines*. Residues involved in the pairs are shown as *spheres*. The *top* residues and pairs are colored based on the discrete clusters they belong to at each coverage cutoff. Increasing coverage shows a growing, highly interconnected network that covers the core of the protein and the GPCR allosteric pathway. The composition of the discrete clusters can be found in Table S7. *A–I*, CovET network formed at various coverage cutoffs in the D2R. *J*, most functional motifs in the D2R are recovered by CovET network at various coverage cutoffs. GPCR, G protein-coupled receptor; TM3, transmembrane helix 3.

To predict covarying residues, we aligned the whole sequence of 2568 class A GPCRs probing a wide cross-section of bioamine receptors and species (Fig. S2*A*). The top-ranked pairs predicted by CovET created a modular network that significantly incorporates experimentally described components of the allosteric pathway extending from the ligand binding site to the G protein coupling and β-arrestin site (*p*-value 3.7E-17 at 30% coverage) (Figs. 4 and S6; Table S6). Moreover, as the network is built-up over a span of CovET ranks, it reveals an underlying modular evolutionary hierarchy that implies various degrees of functional importance. This is because CovET estimates the covariation pattern of residue pairs in the context of the evolutionary tree. The best-ranked CovET pairs have residues that do not vary or if they do, always in concert with the main evolutionary tree branches. As coverage increases, concerted variation tracks with lesser tree branches until, eventually, additional pairs follow no discernable correlation with the phylogenetic tree branches.

Specifically, at only 2.5% coverage, CovET picks up two sets of known functional residues. One of the discrete clusters (Fig. 4*A*, lime, Table S7) overlaps entirely with the NPxxY (67, 68, 70, 72, 74, 78) motif (*p*-value 3.34E-6), while both clusters partially recover the water channel (72) (*p*-value 3.83E-7), the Na^+^ binding site (67, 68, 72, 74, 78) (*p*-value 1E-4), and known state determinants (70, 78) (Fig. S7). At 5% coverage, CovET further adds three discrete clusters, one of which (Fig. 4*B*, *red*) overlaps with the CWxP (68, 70, 78) motif (*p*-value 0.006), and the canonical toggle switch (67) (*p*-value 0.012). At 7.5% coverage, CovET recovers the HHM (67, 68, 72) (*p*-value 0.014) and ionic lock (68, 70) (*p*-value 0.027) as well as switches (68, 70) involved in the allosteric activation pathway (*p*-value 0.001). Two of the prior clusters (Fig. 4*B*, *blue* and *light blue*) now merge and expand. As a result, nearly the entire structurally central and functionally pivotal transmembrane helix 3 (TM3) is recovered (Figs. 4*C*, *blue* and S7). At 10% coverage, CovET predictions significantly recover the DR[E]Y motif (67, 70, 72, 73, 74, 78) and ligand binding site (74, 79, 80, 81, 82) (*p*-value 0.026 and 0.024), while the four discrete clusters are maintained, and the one centered around TM3 continues to expand. At 12% coverage, the cluster including the CWxP motif becomes connected to the central TM3 cluster (Fig. 4*E*, blue). At 14% coverage, CovET recovers the PIF[W] (70, 78) motif (*p*-value 0.05), and the NPxxY motif cluster joins the large network cluster centered on TM3. At 16% coverage, two main clusters are now formed. The larger one spans from TM3 to TM7, while the smaller one is localized between TM1 and TM2. At that coverage, CovET recovers residues from almost all motifs and conserved elements that have been implicated in allosteric signal transduction (*p*-value 1.21E-12), except the TM3-7 (68) lock (not recovered by 30% coverage) and the G-protein coupling site (recovered at 30% coverage, *p*-value 0.028). At 16% coverage, 42 residues are identified by CovET, 31 (∼74%) of which overlap with the ligand or G protein–binding sites, known motifs, state determinants, or members of the allosteric network. Interestingly, some residues are not described among previously recognized functional positions. These include: 65, 75^2.45^, 89^2.59^, 97, 100, 107^3.25^, 139, 160^4.50^, 191^5.40^, 192^5.41^, and 198^5.47^ (superscripts refer to the Ballesteros-Weinstein numbering system for GPCR TM segments) (83). Residues 97, 100, and 107^3.25^ form a small local cluster (Cβ within 8 Å) in the structure, while the hydroxyl group in Ser75^2.45^ is hydrogen bonded with the side chain of Trp160^4.50^ (Fig. S8). Thus, these additional CovET residues are not randomly distributed in the structure and would be of interest for further mutational testing. The remaining two clusters continue to grow at 20% coverage and merge into a single connected network, spanning the 7TM from end to end at 30% coverage. This ranked order of appearance of functional modules that grow and coalesce to span the entire known allosteric pathway is in sharp contrast to the interaction networks identified by DCA, EVCouplings, and ET-MIp which localize to TM1 and do not overlap significantly with key sites (Fig. S6 and Table S6). Together, these results show that CovET recovers hierarchically and specifically the structural and functional motifs most critical components to the allosteric pathway mediating signal transduction in Class A bioamine GPCR.

### Pairs predicted by CovET correlate with epistatic interactions

**Figure 5:**
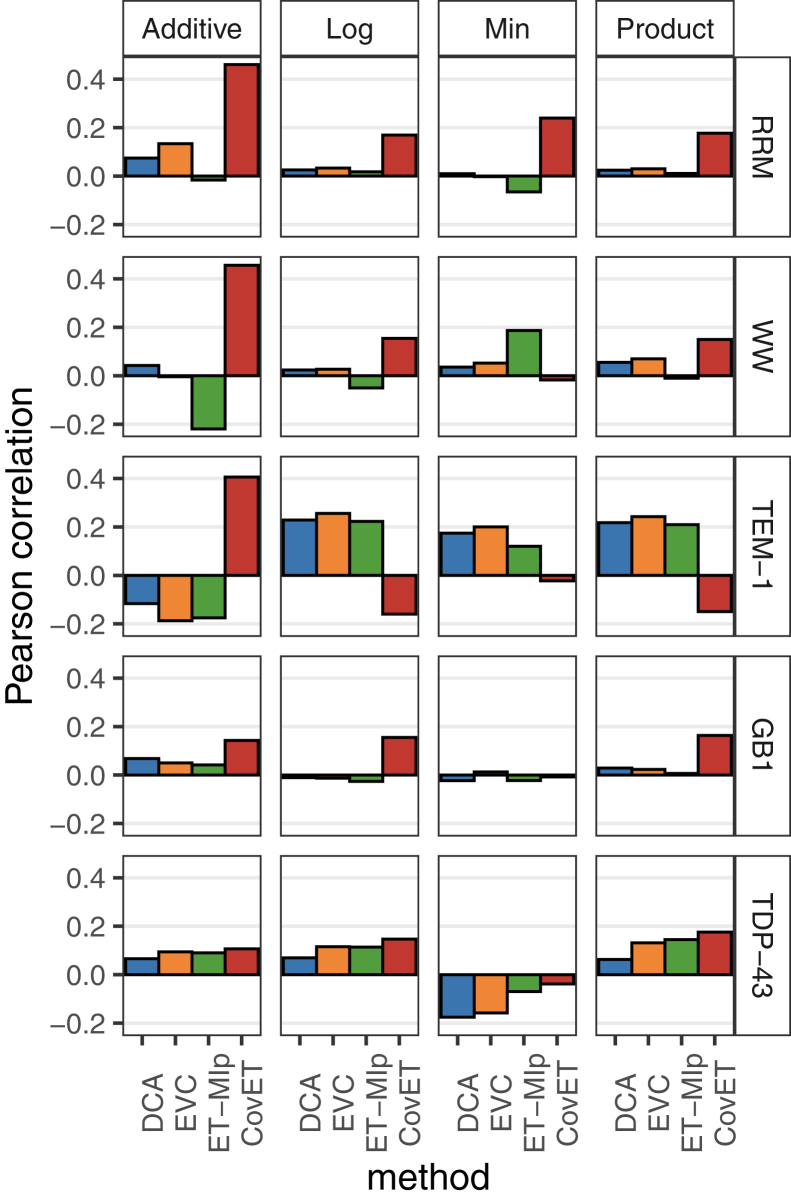
**Correlation of covariation scores with epistasis scores for three proteins.** Epistasis scores were calculated using four different models, and the correlation between the calculated and the average displacement from wildtype was calculated for each pair. These values and covariation scores from the DCA, EVcouplings (EVC), ET-MIp, and CovET methods were measured for their Pearson correlation. For both domains, the evaluated epistasis models were Min, Log, Product, and Additive. The negative raw score for CovET was used because unlike other methods, the higher CovET raw score means a less coupled pair. GB1, IgG-binding domain of protein G; RRM, RNA recognition motif; TAR DNA, prion-like domain of TDP-43.

To test if CovET predicts intraprotein epistatic interactions, we evaluated the correlation of the top predictions with large-scale mutational studies. We analyzed five high-throughput deep mutagenesis studies that measured the fitness of double mutants: the RRM domain (50), the WW domain (84), TEM-1 β-lactamase (85), the IgG-binding domain of protein G (GB1) (86), and the prion-like domain of TDP-43 (TAR DNA-binding protein 43) (87). Because the best epistasis model is unknown, we computed epistasis scores using four commonly applied models (Additive, Log, Min, and Product) (84) for each of the datasets. Since CovET and other covariation methods tested here do not consider the impact of specific mutations pairs and have no directionality, we took the mean and absolute value of all epistasis scores available for a given residue pair, resulting in a final score that represents the average deviation from wildtype behavior. In the RRM dataset, CovET predictions correlate the best with these experimental epistasis scores for all four commonly applied epistasis models (Pearson correlations per epistasis model: Min 0.239, Log 0.170, Product 0.177, Additive 0.460, Figure 5). The next best correlations, from EVCouplings, were much lower across every epistasis model (Fig. 5). The WW domain dataset consisted of 47,000 variants, including 5010 double mutants (∼2.5% of possible) (84). CovET correlated best with the experimentally determined epistasis scores using Log, Product, and Additive models, with Pearson correlation scores of 0.154, 0.149, and 0.456, respectively. For the minimum epistasis model, only ET-MIp correlates with experimental data with highest Pearson correlation of 0.187. For TEM-1, CovET gives the best correlation in the Additive model (Pearson correlation 0.406), which is also the best correlation among all epistasis models. In GB-1 and TDP-43, CovET correlates the best with epistasis scores computed using Additive, Log, and Product models, while none of the covariation methods correlates with the Min epistasis model. In addition, when considering all four epistasis models together, CovET best correlates with experimental data in all five proteins tested (Fig. 5, Pearson correlations: RRM Additive 0.460, WW Additive 0.456, TEM-1 Additive 0.406, GB-1 Product 0.163, and TDP-43 Product 0.176). These results suggested that CovET better predicts protein residue functional couplings than DCA, EVCouplings, and ET-MIp and suggests that phylogenetic couplings are generally in better agreement with Additive epistasis.

## Discussion

Our study introduces a new method to identify evolutionarily coupled sequence positions. CovET is distinct from existing methods (13, 14) in two ways: first, it explicitly accounts for phylogenetic history by applying the ET framework to score every pair of residues recursively along successive evolutionary tree partitions. Second, CovET exclusively penalizes uncoupled variations within a partition’s branch, and pairs that are completely conserved or that covary within a branch are not penalized. We find that, unlike other methods, top CovET predictions form significant spatial clusters that overlap with ligand binding sites in a diverse set of protein families, as well as known functional sites in the RRM and WW domains and in the D2R. The functional relevance of top CovET pairs is further supported by deep mutational scans where CovET predicted epistatic interactions between residues better than other methods. Moreover, the structural clusters and networks defined by top-ranked CovET pairs are functionally relevant. In the D2R, for example, top CovET residues form dense clusters of mutually coupled pairs that overlap with key functional sites and reveal, at increasing coverage, nearly the entire canonical allosteric network. The hierarchical nature of this network, reminiscent of past ET analysis (36), suggests that CovET captures the functional and evolutionary architecture of the Class A GPCR transduction mechanism through couplings that define the allosteric pathway. Given how well CovET recovers the Class A GPCR allosteric activation pathway, it would be interesting to test the couplings predicted by CovET in the D2R. In addition, in our recent study, residues 180 and 181 in the metabotropic glutamate receptor 4 were predicted to be highly covariant by CovET. Indeed, residue position 180 and 181 demonstrated a strong epistatic interaction toward ligand binding in metabotropic glutamate receptor 4 (88). Similar experiments can be conducted on highly ranked CovET residues in the D2R. Further validating our coupling predictions in the D2R *in vitro* or *in vivo* could potentially strengthen our understanding of its physiologically (71, 72, 74, 78) and therapeutically relevant (70, 72, 74, 78) allosteric activation pathway.

The performance of CovET is rooted in the phylogenetic logic of the ET framework. Evolution proceeds from random sequence variations followed by functional selection, repeated at each generation (89, 90, 91). Therefore, the pressure for two residues to comutate, or not mutate at all, will be large when the interaction between the residues maintains an important protein function within a narrow range of fitness tolerance. Conversely, that pressure will be small, or nil, if a large change in protein function is well tolerated by an organism in its new adopted environment. Thus, covariation patterns between two sequences in a protein family must be interpreted with respect to the function each protein serves in its environment. While direct functional assessments of proteins are sparse, the ET approach instead uses distances among phylogenetic branches since these are defined through a sequence metric that approximates functional aptitude groupings (17, 89, 90). As a result, nonconcerted variations across short evolutionary ranges, where function is likely preserved, should strongly indicate a lack of coupling and be penalized more severely than nonconcerted variations across long ranges in the tree. CovET captures the functional context of covariation patterns by scoring the complete alignment at the tree’s root node and each subalignment spawned by a divergence event. In contrast: traditional covariation methods are performed over a complete alignment, without consideration of phylogenetic structure (92). Such an approach intrinsically assumes that all sequences share the same structure and function, which is less and less likely as we consider proteins that are further and further apart in sequence identity and in an evolutionary tree. These methods may then be confounded by less meaningful covariations in functionally unrelated regions of the tree. In addition, most covariation models penalize pairs of residues that are invariant together (93), even though, presumably, many of these important positions are essential for protein function. CovET considers both conservation and covariation as positive signals and penalizes neither. By exclusively penalizing nonconcerted variations—the only signal that one can be sure does not support covariation—CovET avoids uncertainties regarding invariant and mostly invariant residues.

Given its unique approach to identifying coupled residues, CovET may provide an orthogonal feature set to current covariation methods in protein structure prediction machine learning systems (13, 14). CovET nearly recapitulates the performance of other methods in predicting structural contacts, but its predictions are fundamentally unique, they are further apart in sequence, yet significantly spatially clustered. CovET may also be used to improve genotype-phenotype predictions. Taking a more formal approach, we previously used ET to approximate the first derivative of the evolutionary landscape function f(γ)= φ, which maps genotypes γ to their fitness potential φ (94). This approach allowed us to estimate the functional impact of coding variants with high accuracy (95) and was further validated with diverse practical applications (96, 97, 98, 99, 100, 101). However, ET only evaluates the functional importance of single residues, thus missing higher order interactions among residues. CovET may be interpreted as the mixed second derivative of f with respect to residue pairs. In the future, with proper scaling and sign, the addition of this second-order epistatic term may improve our approximation of the evolutionary landscape function and our understanding of the genotype-phenotype relationship.

In summary, CovET predicts functionally coupled sequence positions by accounting explicitly for phylogenetic divergences during evolution. This approach enriches current views of residue couplings by informing whether variants occur in preserved or divergent functional contexts. Examples from functional sites and an allosteric pathway suggest this approach may provide additional insights to understand protein structure and function and machine learning features to predict them.

## Experimental procedures

### Sequence retrieval and multiple sequence alignment construction

To test our algorithm on the most diverse set of proteins possible, we turned to the Pfam database (42). The protein families used in this study are summarized in Table S1. The Pfam database contains a comprehensive list of protein families, each represented by a multiple sequence alignment. We extracted alignments for each family and removed those where no family member had an available experimental structure with at least 3.5 Å resolution. In the case where multiple structures were available for a family, the structure that best aligned to its respective linear sequence was assigned as the reference structure for the family; its linear sequence was assigned the query sequence. Alignments were then filtered using hhblits (102) to minimum 70% coverage with query, minimum 30% sequence similarity to query, and 98% maximum pairwise sequence similarity. Larger alignments were filtered to contain the most diverse 2000 sequences. All filtering steps were done in tandem using the command: hhfilter -i <alignment_path> -cov 70 -qid 30 -id 98 -diff 2000. Due to the presence of small proteins with few contacts (*e.g.*, single α helices), families were further filtered to have at least one short-range (6–11 amino acids apart in sequence), medium-range (12–24 amino acids apart in sequence), and long-range (24+ amino acids apart in sequence) structural contact (103, 104). The Pfam database is further grouped into clans of families that share an evolutionary origin. To ensure coverage of the entire known proteome, for each clan in the Pfam database that has at least one family that meets the above criteria, we used the family with the highest number of sequences to perform CovET and other methods.

The RRM domain (105), the WW domain (60), the D2R (70, 78), TEM-1 β-lactamase (85), the GB1 (86) and the prion-like domain of TDP-43 (87) were used as detailed examples in this study (Table S2). Homologous sequences for these proteins were retrieved by using the blastp utility of the BLAST+ tool (106) to search the UniProt90 sequence database (downloaded from https://www.uniprot.org/downloads) (107). This sequence database was first filtered to remove all sequences including the terms “Fragment” or “Low Quality”. Then, it was used to create a database compatible with the blastp tool using the makeblastdb utility also provided with the BLAST+ tool. Blastp (version 2.9.0, build May 27, 2019) was run using the filtered database as the target and a max e-value cutoff of 0.05 (41) and a max sequence return of 20,000. Sequences identified by the BLAST search were further filtered such that only sequences covering at least 70% of their query and with identity of 25% ≤ query ≤ 98% (41, 89) were kept. Sequences were also removed if they included amino acids other than the standard 20 or gaps, if their description included the terms “artificial”, “fragment”, “low quality”, “partial”, or “synthetic”, or if their taxonomy recorded in UniProt included the terms “synthetic” or “artificial”. The sequences passing these filters were aligned using the ClustalW tool (version 2.1) (47, 108) using the quicktree option. To reduce redundant sequences, all pairwise identities were calculated between sequences in the constructed alignments. Sequences were removed such that any cluster of sequences with >98% sequence identity to each other were represented by only one sequence. The alignment was re-aligned using ClustalW with the same settings after this filtering step.

### Phylogenetic reconstruction under ET framework

In the ET framework (17, 41, 109), the distance between any two sequences in the multiple sequence alignment is computed by:

(1) $$Dist\left({seq}_{a},{seq}_{b}\right)=1\;-\;\frac{\sum_{i=1}^{l}f\left({seq}_{a,i},{seq2}_{b,i}\right)}{\min\left(\sum_{i=1}^{l}g\left({seq}_{a,i}\right),\sum_{i=1}^{l}g\left({seq}_{b,i}\right)\right)}$$

where ${seq}_{a}$ is the protein sequence at the a th row in the multiple sequence alignment. ${seq}_{a,i}$ is the amino acid in the i th position in a th sequence (character at [a, i] when considering MSA as a matrix), and l is the column count of the MSA.

$$f\left(x,y\right)=\left\{ \begin{array}{c} 1,if\;BLOSUM62\left(x,y\right)\geq 2\\ 0,if\;BLOSUM62\left(x,y\right)<2 \end{array}\right.$$

where BLOSUM62(x,y) is the log odds of between amino acid character x and y in the BLOSUM62 matrix. The log odds between gap character and any character (including itself) are manually set to 0.

$$g\left(x\right)=\left\{ \begin{array}{l} 0,if\;x\;is\;the\;gap\;character\\ 1,otherwise \end{array}\right.$$

A UPGMA tree is generated across the sequences using the distance matrix (110).

### Covariation predictions

CovET is first described here, and the code is available in the accompanying codebase. The formula for the covariation of a pair of positions is given by:

(2) $$CovET_{ij}=1+\sum_{n=1}^{N-1}\frac{1}{n}\sum_{g=1}^{n}e^{-\sum_{v\in NC}f_{ij,v}\;\ln\left(f_{ij,v}\right)}$$

$$v_{ab,ij}\equiv \left(x_{a,i},x_{a,j},x_{b,i},x_{b,j}\right)$$

$$x_{a,i},x_{a,j},x_{b,i},x_{b,j}\in \left\{20\;animo\;acids,gap\right\}$$

$$v_{ab,ij}\in NC,if\;\left(x_{a,i}=x_{b,i}\;\&\;x_{a,j}\neq x_{b,j}\right)\;or\;\left(x_{a,i}\neq x_{b,i}\;\&\;x_{a,j}=x_{b,j}\right)$$

where ij is a pair of residue positions, N is the depth of the phylogenetic tree, n is a level in the phylogenetic tree, and g is a group of sequences (branch) at that level in the tree. The term in the second sum is the diversity metric or perplexity, which is the exponential of the Shannon entropy. When comparing a pair of residues in two sequences in a 21-letter alphabet for all amino acids and a gap character, there are 194,481 (21^4^) possible outcomes for the 20 standard amino acids plus a gap character. These possible outcomes can be classified as conservation (AD > AD), concerted variation (AD > CE), and nonconcerted variation (AB > AE). Four hundred one (21^2^) of them are conservations. 176,400 (21 × 21 × 20 × 20) of them are concerted variation. The remaining 17,640 of them are nonconcerted variations $\alpha^{2}\times 2\left(\alpha -1\right)$, respectively, where α is the number of characters used. v in the entropy calculation is any of the 17,640 possible non-concerted variations (NC). The process of characterizing a pair of positions for a given group, g, is demonstrated in Figure 1. By scoring these transitions, nonconcerted variation is penalized, while conservation and concerted variation, or covariation, are not.

ET-MIp (16) was reimplemented in Python and is available with the codebase distributed with this study. ET-MIp score for a pair of residues are given by the equation:

(3) $$ETMI_{p,ij}=\sum_{n=1}^{N-1}\frac{1}{n}\sum_{g=1}^{n}MI_{p}^{g}\left(i,j\right)$$

where ij is a pair of residues, N is the depth of the phylogenetic tree, n is a level in the phylogenetic tree, and g is a group of sequences (branch) at that level in the tree. $MI_{p}^{g}\left(i,j\right)$ is mutual information with the product correction (111) given by:

(4) $$MI_{p}\left(i,j\right)=MI\left(i,j\right)-APC\left(i,j\right)$$

where MI(i,j) is the mutual information (112):

(5) $$MI\left(i,j\right)=H_{i}+H_{j}-H_{ij}$$

where $H_{i}$ and $H_{j}$ are the Shannon entropy of positions i and j, respectively, and $H_{ij}$ is the joint entropy of the pair of position. APC(i,j) is the average product correction (111) given by:

(6) $$APC\left(i,j\right)=\frac{MI\left(i,\bar{y}\right)\cdot MI\left(j,\bar{y}\right)}{\bar{MI}}$$

where $MI\left(i,\bar{y}\right)$ and $MI\left(j,\bar{y}\right)$ are the mean MI with respect to position i or j, and $\bar{MI}$ is the mean mutual information over the pair of positions.

EVCouplings (13, 43) covariation scores were computed using the implementation provided at https://github.com/debbiemarkslab/EVcouplings. To perform EVcouplings predictions, the provided config file was used to set the following settings for each run. In the “global” settings: “region” = None, “theta” = 0.8. The “pipeline” was set to “protein_monomer”. “batch” was set to None. The “stages” were set to [“align”, “couplings”]. For the “align” settings: “protocol” = “existing”, “input_alignment” was set to the path of the alignment file used by all prediction methods, “first_index” = 1, “compute_num_effective_seqs” = False, “seq_id_filter” = None, “minimum_sequence_coverage” = 0, “minimum_column_coverage” = 0, “extract_annotation” = False. These settings were used to ensure that the provided alignment was used without alteration, so that results would be comparable across methods. For the “couplings” settings: “protocol” = “standard”, “iterations” = 100, “lambda_J” = 0.01, “lambda_J_times_Lq” = True, “lambda_h” = 0.01, “lambda_group” = None, “scale_clusters” = None, “alphabet” was set to the ‘-‘ gap character and the 20 standard amino acids, “ignore_gaps” = False, “reuse_ecs” = True, and “min_sequence_distance” = 0. The “global” variables: “prefix”, “sequence_id”, and “sequence_file” were provided separately for each protein.

DCA was computed using the multivariate Gaussian approach implemented in Julia (44) and available at https://github.com/carlobaldassi/GaussDCA.jl.

### Evaluation of structural contacts

To evaluate structural contacts, query sequences were first aligned with the corresponding structures (“PDB” column of Table S1), and only positions present in both were considered for evaluation. Contacts were then determined using the definition used in the CASP competitions, only residues at least six residues apart in sequence and whose Cβ (C*α* for glycine) were within 8 Å of each other were considered true contacts (103, 104, 113, 114, 115, 116, 117, 118). Contacts were broken into three categories, also defined in CASP by number of amino acids of separation, which were short (6, 7, 8, 9, 10, 11), medium (12, 13, 14, 15, 16, 17, 18, 19, 20, 21, 22, 23), and long (≥ 23) (103, 104, 117). Finally, predictions were compared against the combined set and three subsets of contacts using the AUROC and AUPRC, as implemented in Sklearn (119). All predicted contacts were evaluated, not just top contacts, as suggested in a recent CASP competition (104). The AUPRC were then adjusted by subtracting the observed positive rate, which is the expected AUPRC for a random predictor. The AUROCs and adjusted AUPRCs of each method were compared using paired two-sided Wilcoxon Rank Sum test to determine if differences were statistically significant.

### Evaluation of structural clustering using the selection cluster weighting z-score

The SCW z-score (41, 45, 46, 47, 120) was used to measure the nonrandomness of top covariation predictions mapped to the protein structure. As described in the previous section, residues were only considered when they could be aligned between the query sequence and target structure. We then converted the covariation rankings into single residue rankings. Each residue was ranked based on the score of the best covariation pair it forms between other residues. SCW z-scores were calculated for the top 30% of residues, a cutoff which has been used in previous studies (45, 46) and has been shown to correspond with the upper limit of clustering significance in previous studies (47). The SCW z-score can be computed using the code distributed with this study or using the PyETViewer plugin for PyMol (121). It is described by the equation:

(7) $$w=\sum_{i<j}^{L}S\left(i\right)S\left(j\right)A\left(i,j\right)b\left(i,j\right)$$

where L is the full set of pairs of residues present in a protein (counted only once per pair as specified by the term i<j), S is a selection function and returns one for a given residue (i or j) if that residue is in the set of pairs described by the 30% coverage cutoff, and A is an adjacency matrix for all residues in the structure where position i,j is one if the shortest distance between atoms of the two residues is <4 Å and 0 otherwise. The term b(i,j) is the bias coefficient, for unbiased analyses b(i,j) evaluates to one for all pairs of residues, while for biased analyses b(i,j) evaluates to the sequence separation between the two residues (*i.e.*, |i−j|). Differences between methods over all the proteins in the Pfam dataset were measured using paired two-sided Wilcoxon Rank Sum test.

### Determination of average sequence separation

To determine the difference in pair biases for each of the covariation methods, the average sequence separation between residues predicted among the top-ranked pairs was determined. Top pairs were selected for evaluation until 30% of the residues in the structure was included. The sequence separation between the residues of the pair was calculated, *i.e.*, if a pair consists of residues i and j, the sequence separation is given by |i−j|. The average of all sequence separations in the set of top pairs for each protein was calculated, and methods were compared using paired two-sided Wilcoxon Rank Sum test to determine if there was a significant difference in the average sequence separations.

### Recovery of functionally important residues

We obtained the coordinates for biological ligands from the BioLiP database (49) for each of our Pfam queries. A residue was considered as functionally important if the distance between any atom is smaller than 4 Å between that residue and the biological ligands. As with the calculation of SCW z-scores, we then converted the covariation rankings into single residue rankings. Each residue was ranked based on the score of the best covariation pair it forms between other residues. Single residue rankings were compared against the set of functional important residues using the AUROC and AUPRC. The AUPRCs were then adjusted by subtracting the observed positive rate, which is the expected AUPRC for a random predictor. The AUROCs and adjusted AUPRCs of each method were compared using paired two-sided Wilcoxon Rank Sum test to determine if differences were statistically significant.

### Scoring of the identification of gold standard residues

Key conserved residues, motifs, functional sites, and other contributors to protein structure-function from the literature were identified for the RRM and WW domains and the D2R. The overlap of covariation predictions at different coverage cutoffs (10, 20, and 30% for the RRM and WW domains and 2.5, 5, 7.5, 10, 12, 14, 16, 20, and 30% for D2R) with these key sites as well as the union of all sites for each domain or protein were measured for significance using the one-sided hypergeometric test. For the WW domain, a set of known variable residues was also evaluated as a negative control.

### Measuring the correlation of covariation predictions with experimental data

Single and double mutant data from large-scale mutagenesis screens of the RRM domain (50), the WW (84) domain, TEM-1 β-lactamase (85), the GB1 (86), and the prion-like domain of TDP-43 (87) were used to compute epistasis scores for these two domains. The log fitness scores (lnW) of GB1 were obtained from Rollins *et. al* (122) and were exponentially transformed back to fitness before the epistasis scores calculation. The uncorrected toxicity scores for double mutants were used for TDP-43 (87). The toxicity scores for double and single mutants were also exponentially transformed back to have the WT toxicity score normalized to 1. Epistasis scores were computed based on the reported fitness values for single and double mutants using four different models of epistasis: Product, Additive, Log, and Min, the formulas for each of these models are provided below (84).

(8) $$\varepsilon_{ab}^{product}=M_{ab}-M_{a}\cdot M_{b}$$

(9) $$\varepsilon_{ab}^{additive}=\left(M_{ab}+WT\right)-\left(M_{a}+M_{b}\right)$$

(10) $$\varepsilon_{ab}^{\log}=M_{ab}-\log_{2}\left(\left(2^{M_{a}}-WT\right)\cdot \left(2^{M_{b}}-WT\right)+WT\right)$$

(11) $$\varepsilon_{ab}^{\min}=M_{ab}-\min\left(M_{a},M_{b}\right)$$

In each of these formulas, $M_{ab}$ is the fitness value measured for double mutant ab, $M_{a}$ is the fitness value of single mutant a, $M_{b}$ is the fitness value measured for single mutant b, andWT is the fitness value measured for the wildtype domain. In these studies, the fitness values were normalized against wildtype fitness so WT is always 1.

Since the covariation metrics only provide a single score for each pair and the experimental studies provide many more, the mean of all epistasis scores for a given pair was taken. Similarly, covariation metrics evaluated here do not provide a sign to their prediction, so the absolute value of the mean calculated for each pair was taken. This means the covariation scores were compared to the average displacement from wildtype activity as measured by each epistasis model. Only pairs tested in the experimental studies were tested, and the Pearson correlation coefficient between the raw score of each covariation method and each set of epistasis scores was computed to determine which method corresponded better with experimental observations. The negative raw score was used for CovET, because the CovET raw score has a different direction wherein a lower value means better covariation between a pair.

## Data availability

The code for CovET is open and freely available on GitHub at: https://github.com/LichtargeLab/Covariation-ET.

## Supporting information

This article contains supporting information (13, 16, 41, 44, 47, 89, 106, 107, 108, 111, 112, 123, 124, 125, 126).

## Conflict of interest

The authors declare no conflict of interest with the contents of this article.
