# Supplementary material for: CovET: A covariation-evolutionary trace method that identifies protein structure–function modules
Source: J Biol Chem. 2023 Jun 7;299(7):104896. doi: 10.1016/j.jbc.2023.104896 (PMC10338321; doi:10.1016/j.jbc.2023.104896)
Supplement: Supporting Figures S1–S8 [file mmc1.docx]

Supporting information for

**CovET: A Covariation-Evolutionary Trace method that Identifies Protein Structure-Function Modules**

Daniel M. Konecki^†^, Spencer Hamrick^†^, Chen Wang (王 忱)^†^, Melina A. Agosto, Theodore G Wensel, Olivier Lichtarge^*^

* To whom correspondence should be addressed. Email: [lichtarge@bcm.edu](mailto:lichtarge@bcm.edu)

^†^ The first 3 authors are joint First Authors.

**This section includes:**

Supplementary Figures 1 to 8

Legends for Supplementary Tables 1 to 7

Additional References


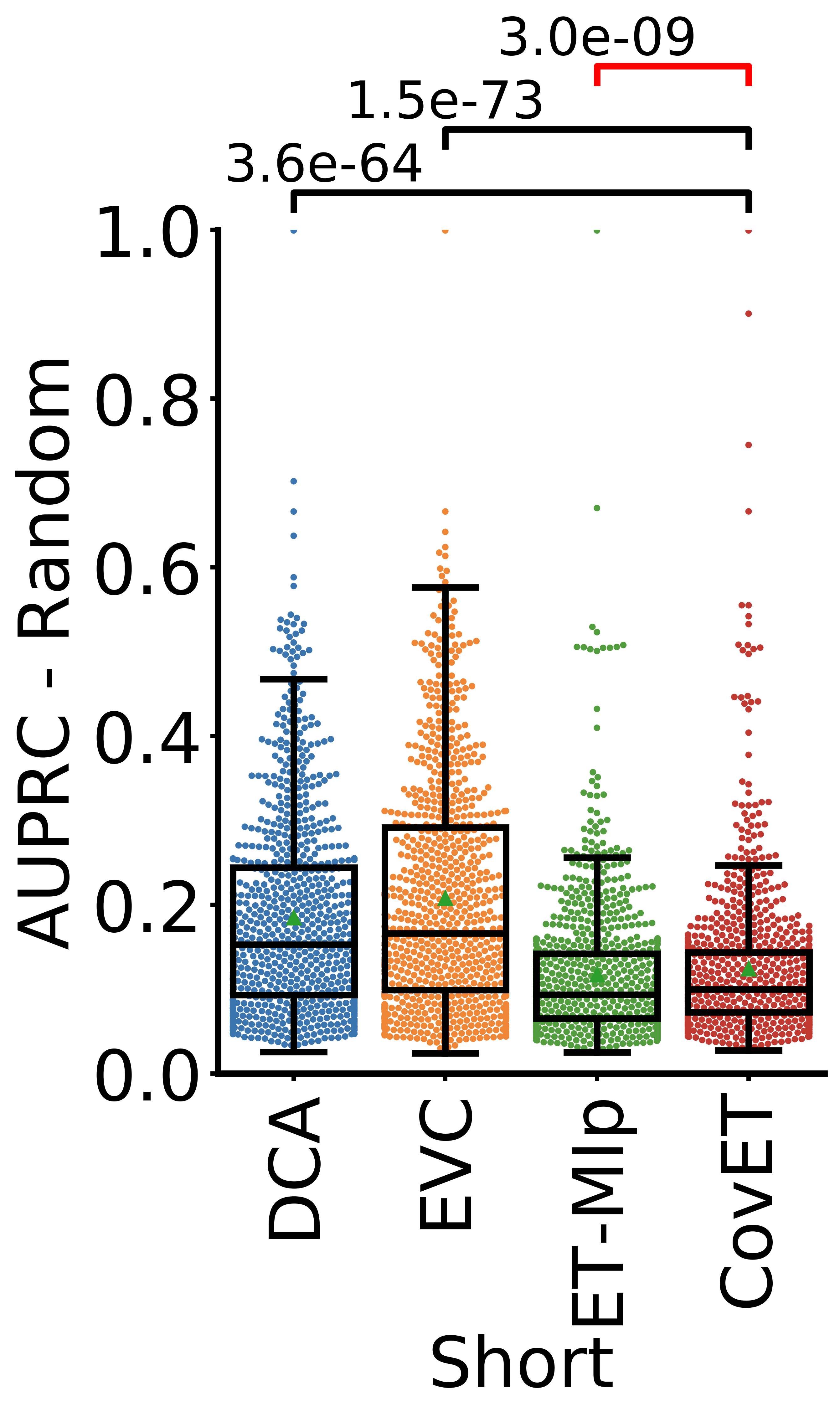

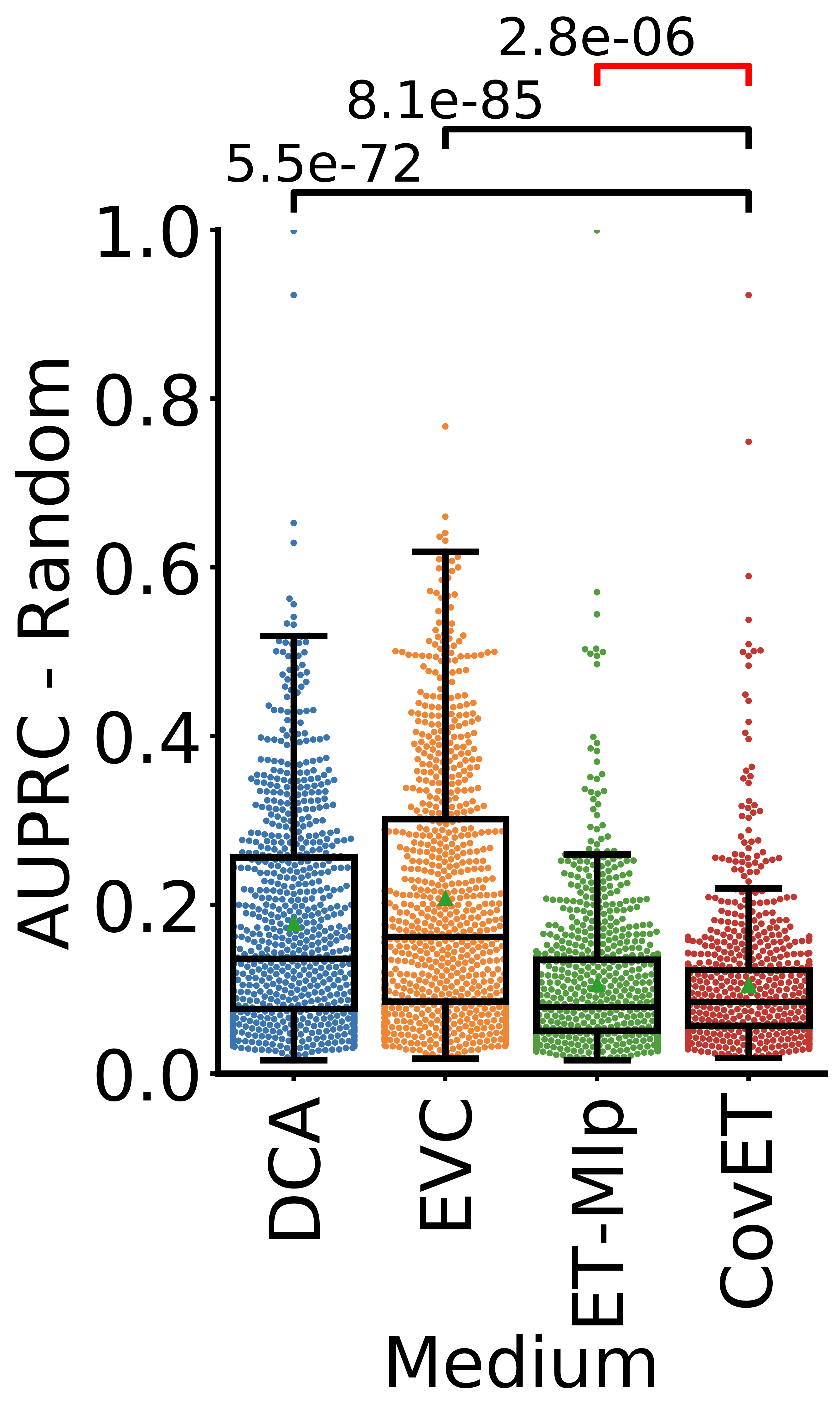

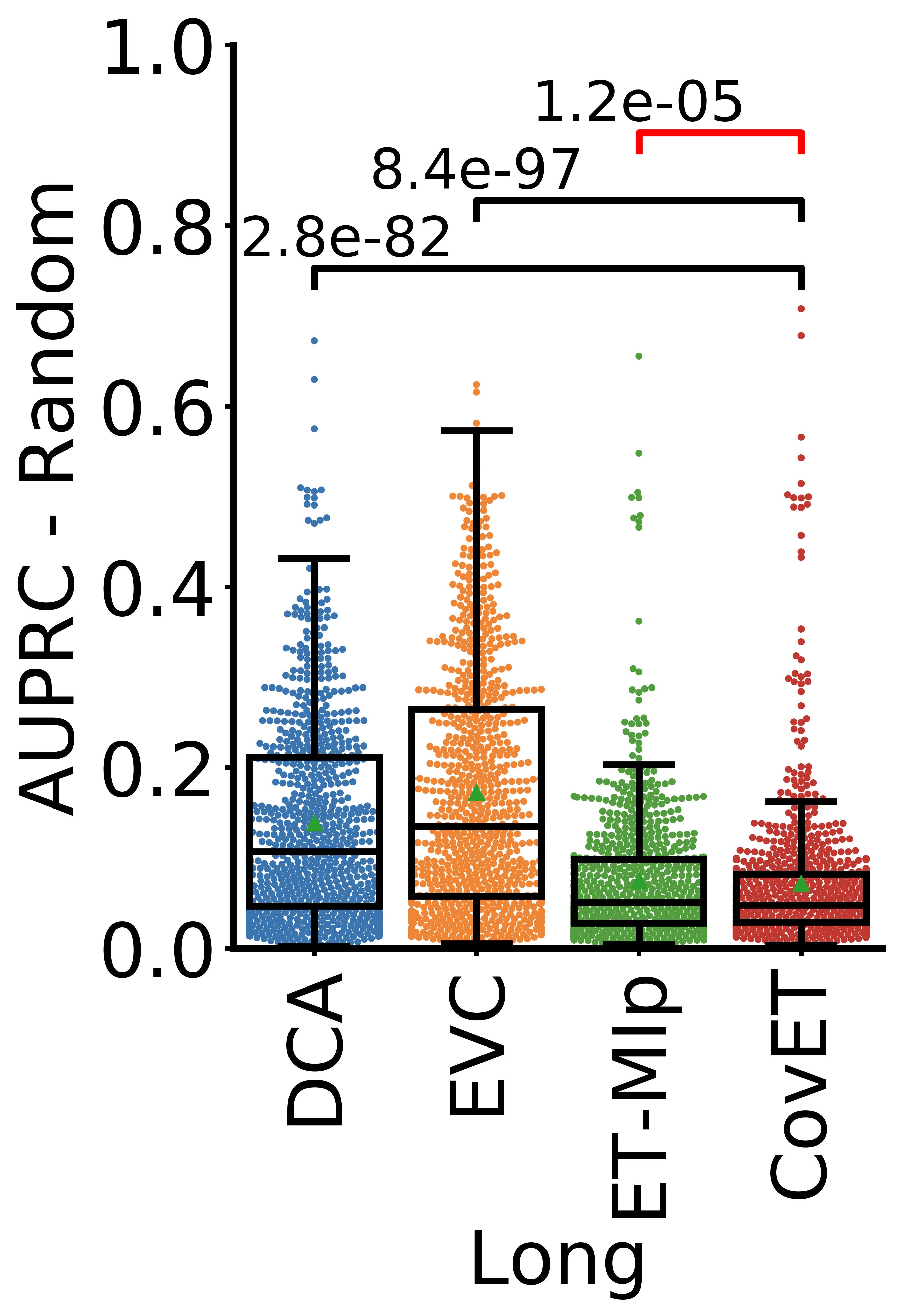

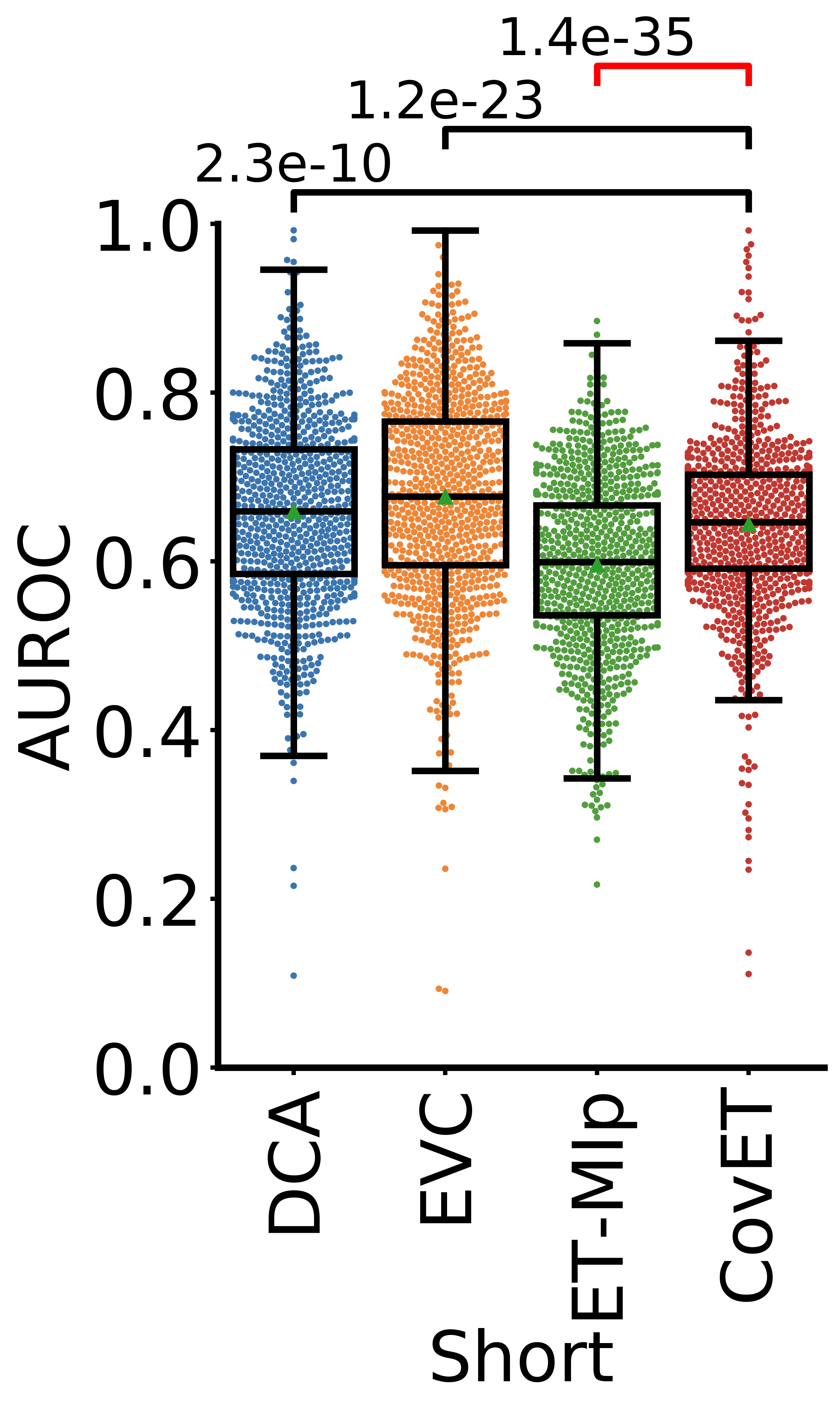

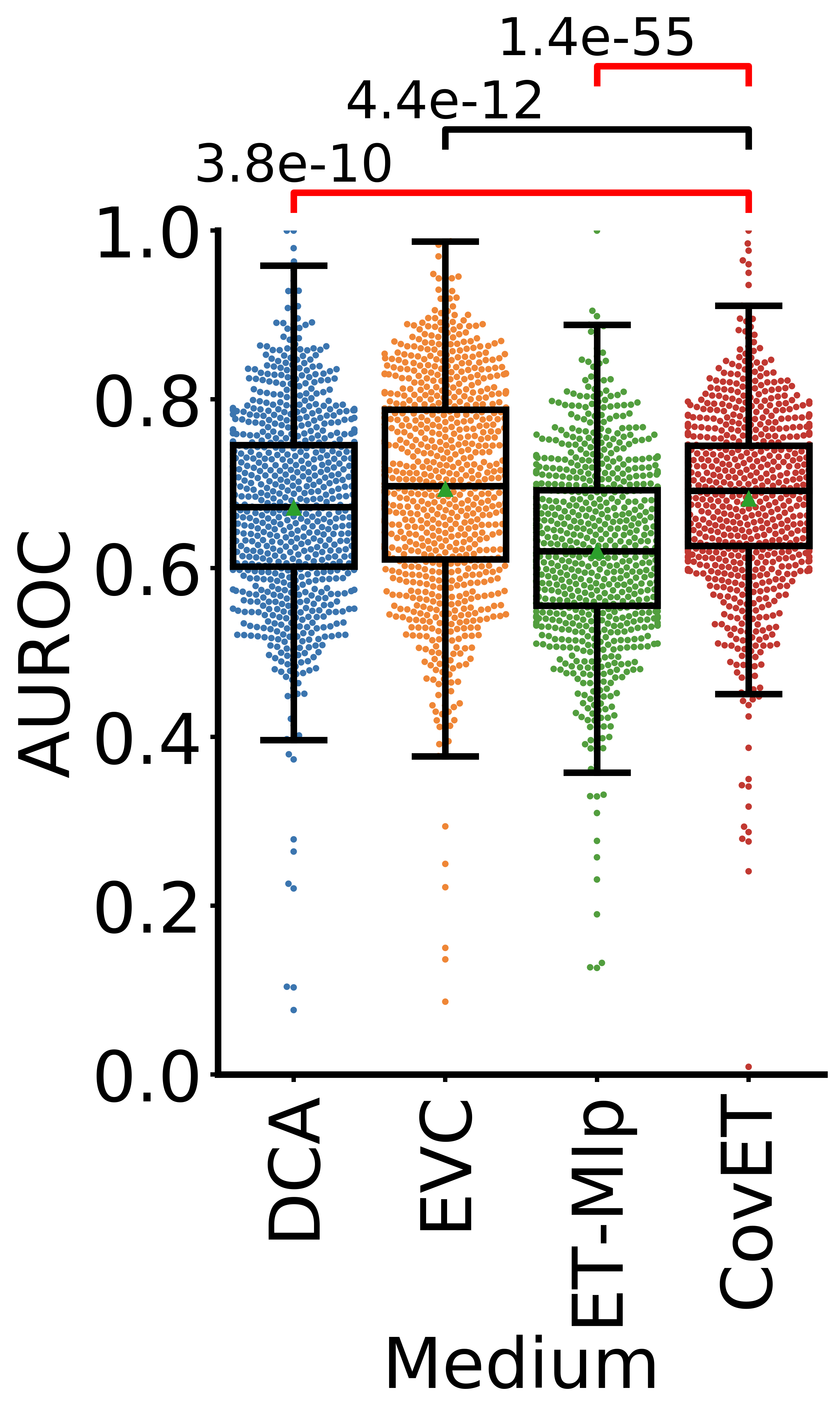

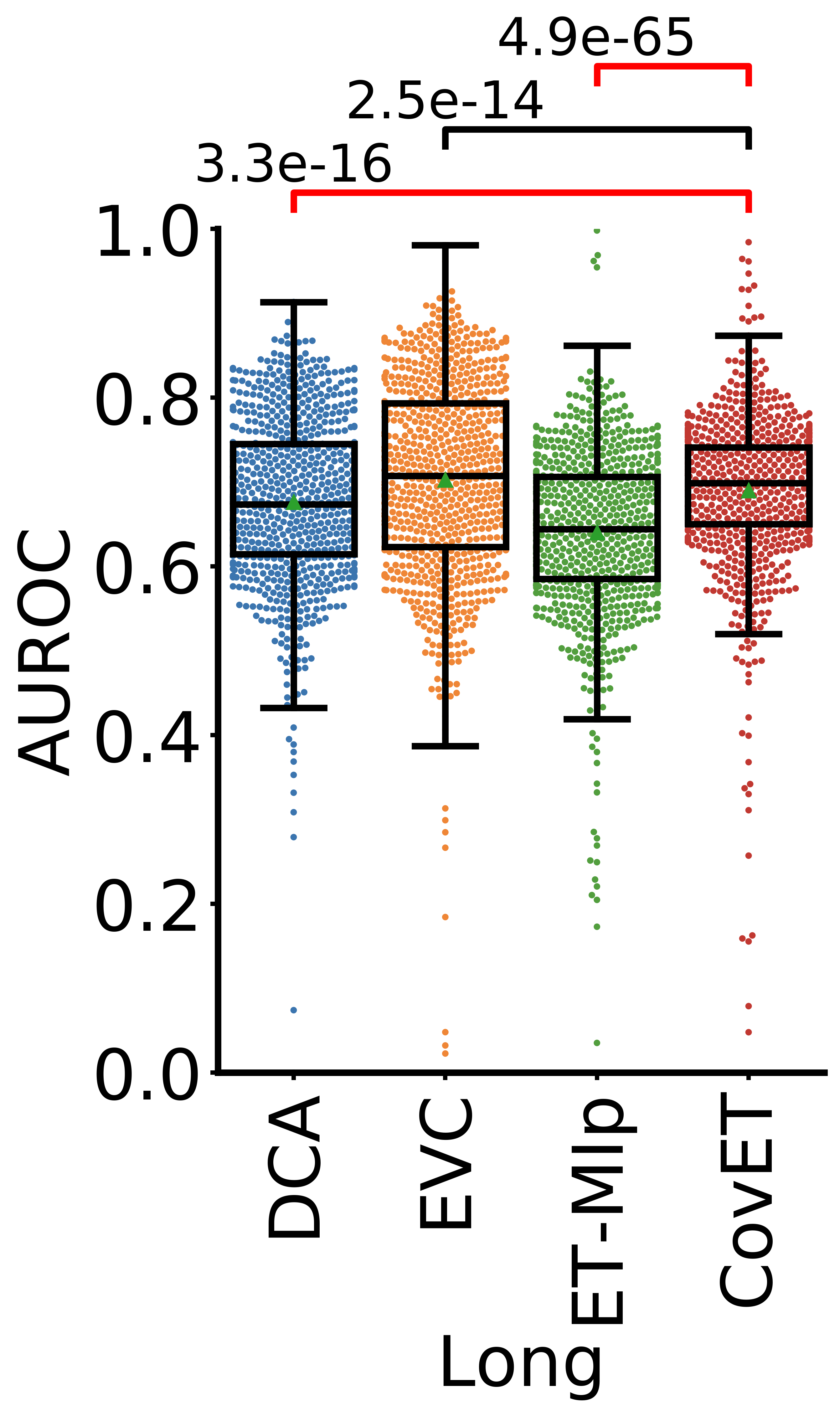


**Supplementary Figure 1. AUROC and AUPRC for recovery of direct contact in Pfam dataset.** Contacts were classified as (residues where Cβ are within 8Å of each other, C⍺ for glycine) at short (6-11 residues apart), medium (12-24 residues apart), and long (>24 residues apart) range sequence separation. CovET outperforms DCA in medium and long-range contacts prediction judging by AUROC, but was outperformed by EVC.


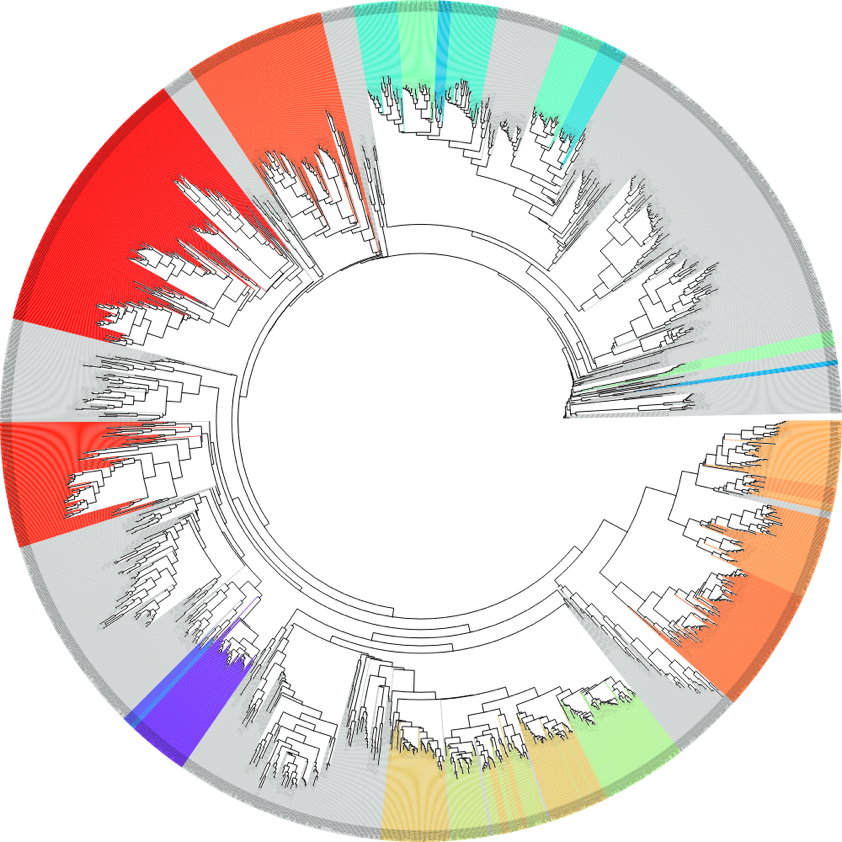

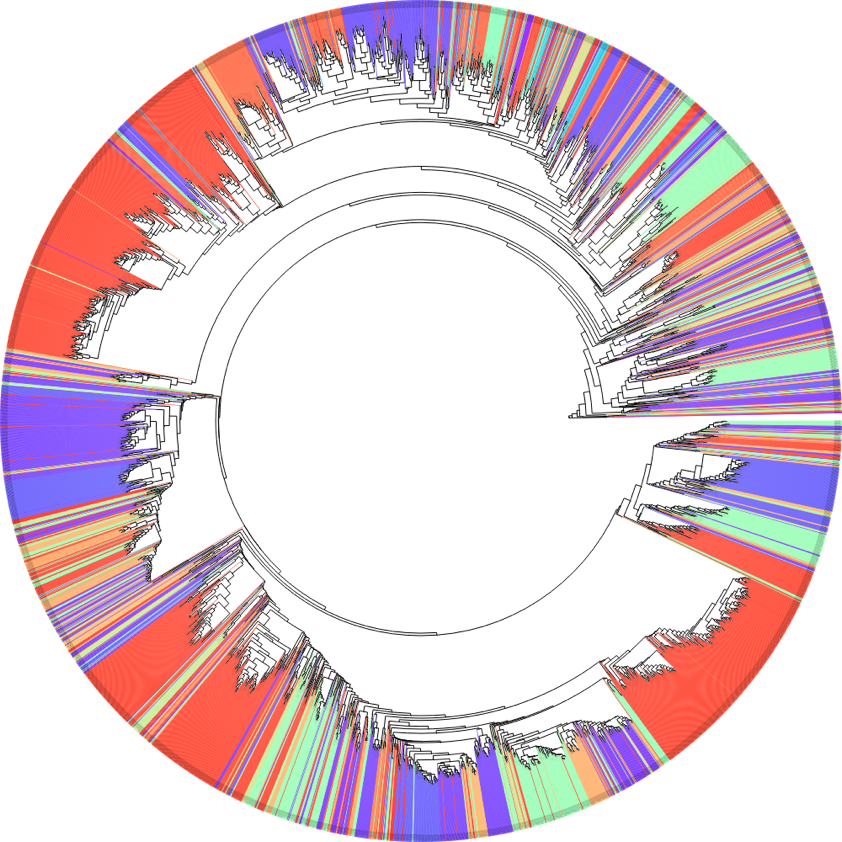

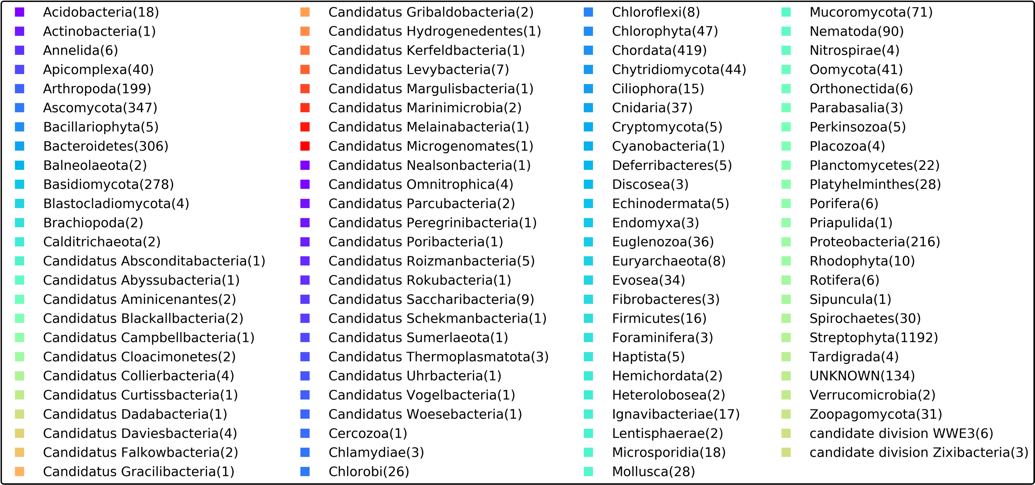

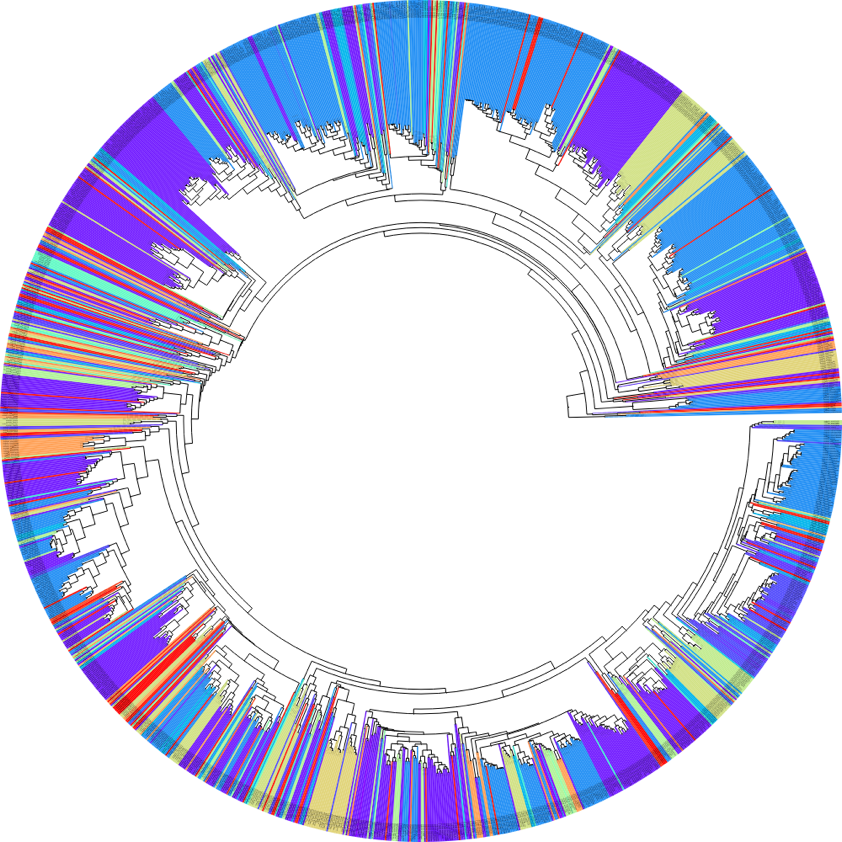

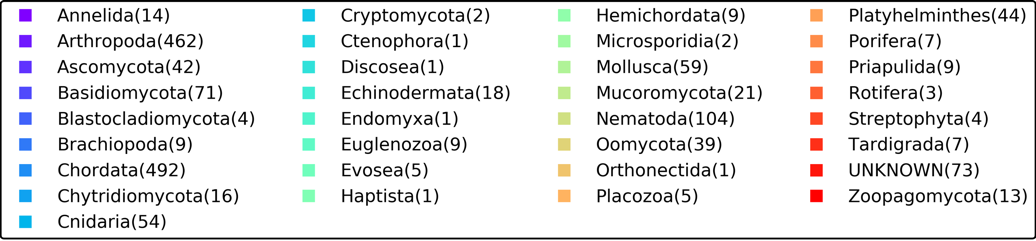


A

B

C


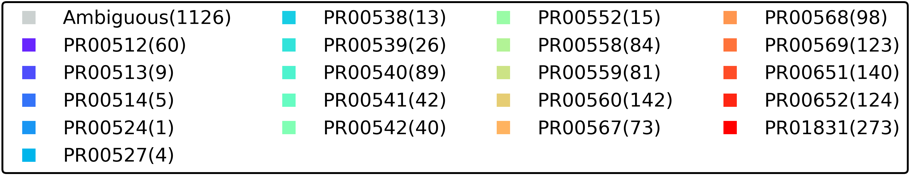


**Supplementary Figure 2. Phylogenetic Trees of 3 proteins show coverage of wide range of protein classifications and species.** Renderings of the phylogenetic trees computed from the alignments of the three proteins studied in greater depth. (A) The phylogenetic tree for the D2 Dopamine Receptor, leaves are colored according to the most specific PRINTS database annotation available for the corresponding UniProt entry. The query sequence is labeled as PR00567 (DOPAMINED2R). (B) The phylogenetic tree for RRM1-2 of PAB, leaves are colored according to the phylum of the corresponding UniProt entry. The query sequence is labeled Ascomycota. C) The phylogenetic tree for the WW Domain of human YAP2, leaves are colored according to the phylum of the corresponding UniProt entry. The query sequence is labeled Chordata.

AUPRC

AUROC


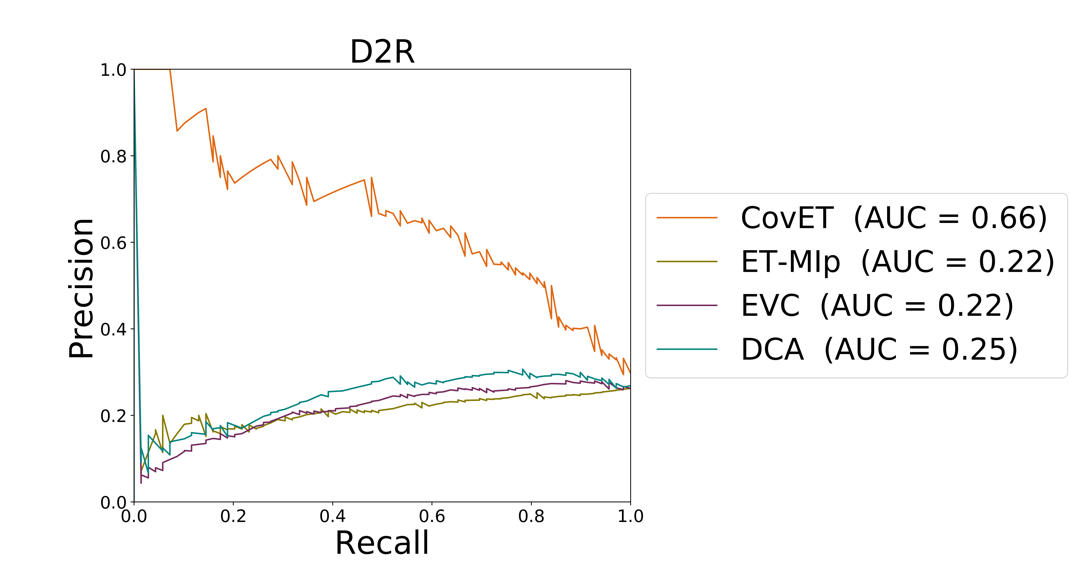

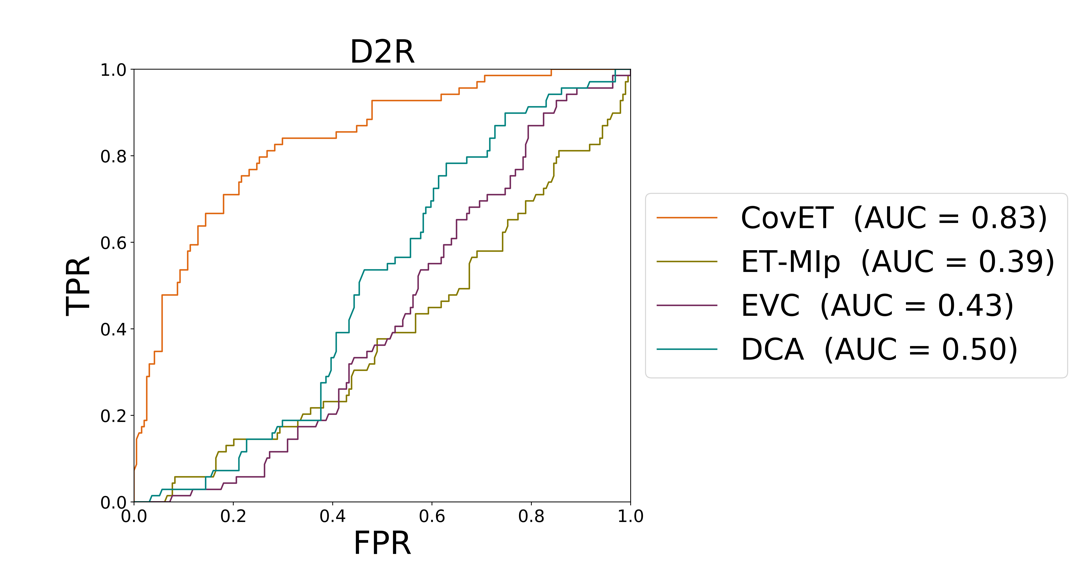

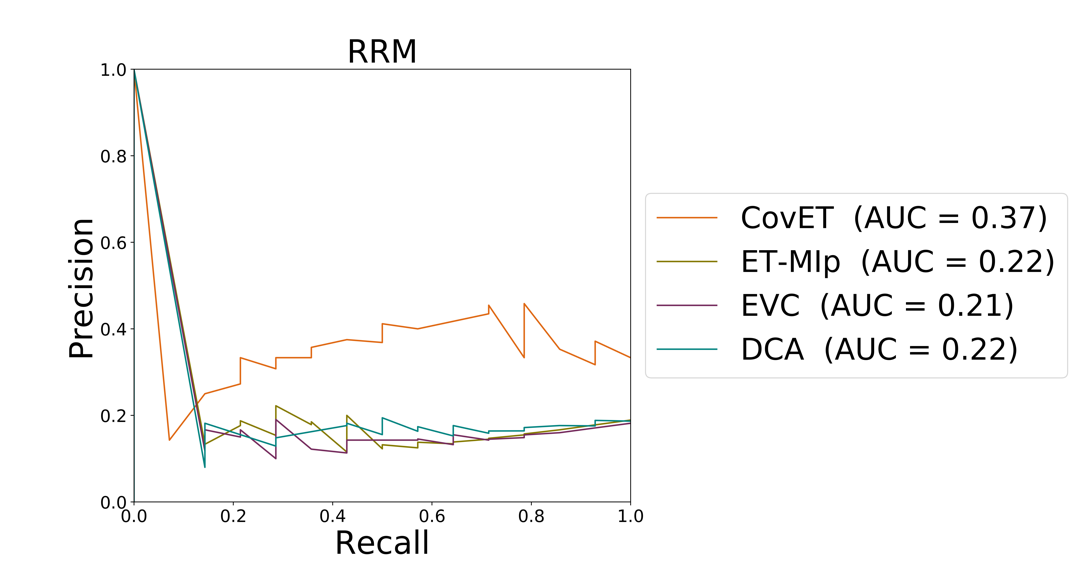

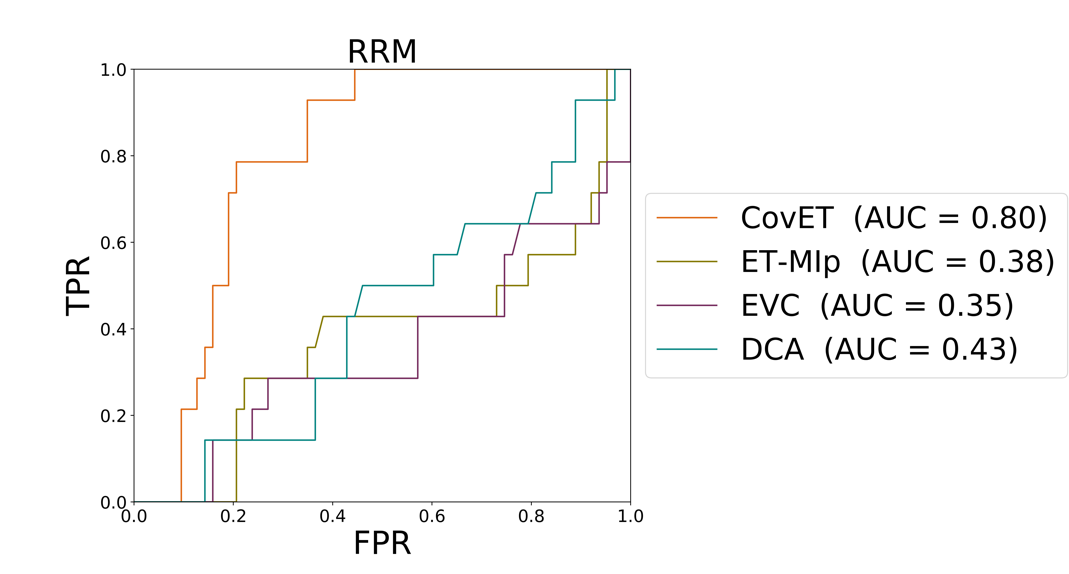

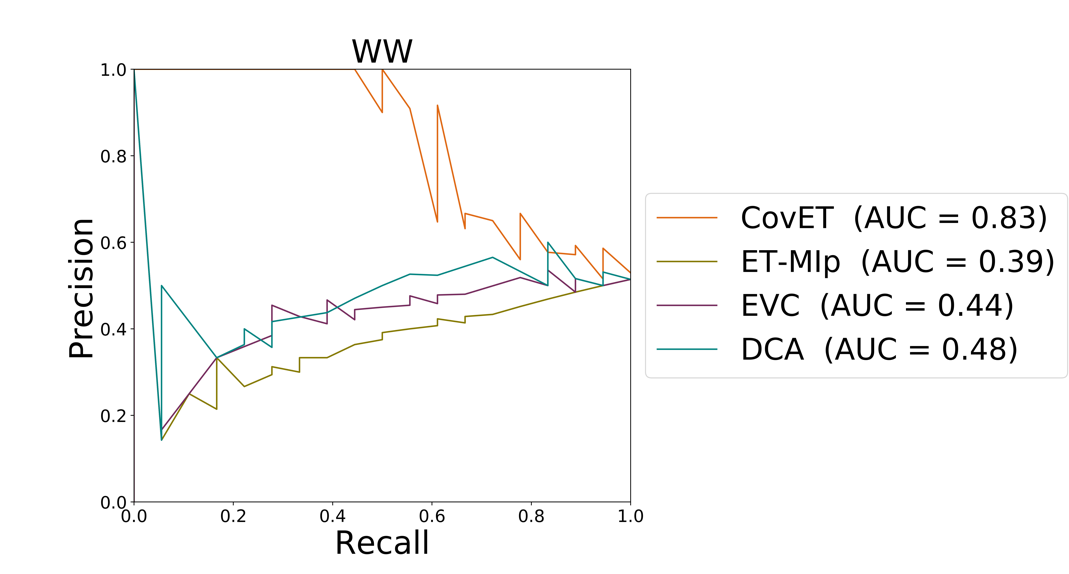

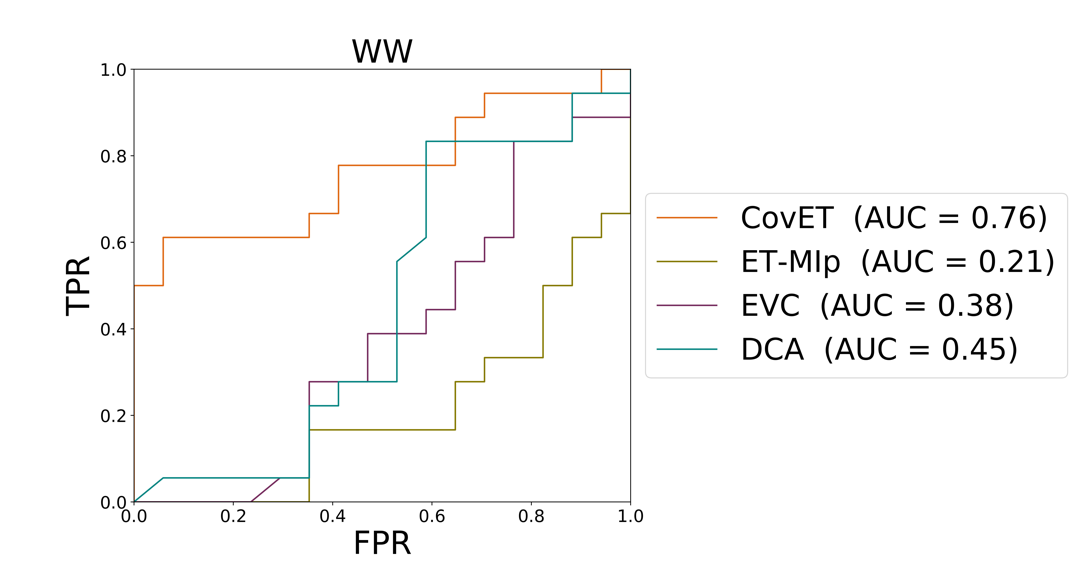


**Supplementary Figure 3. AUROC and AUPRC Measurements Show That Top CovET Predictions Recover Key Residues in the RRM and WW domains and Dopamine D2 Receptor.**

30% Coverage

20% Coverage

10% Coverage

EVC

ET-MIp

CovET

DCA

A

B

C

D


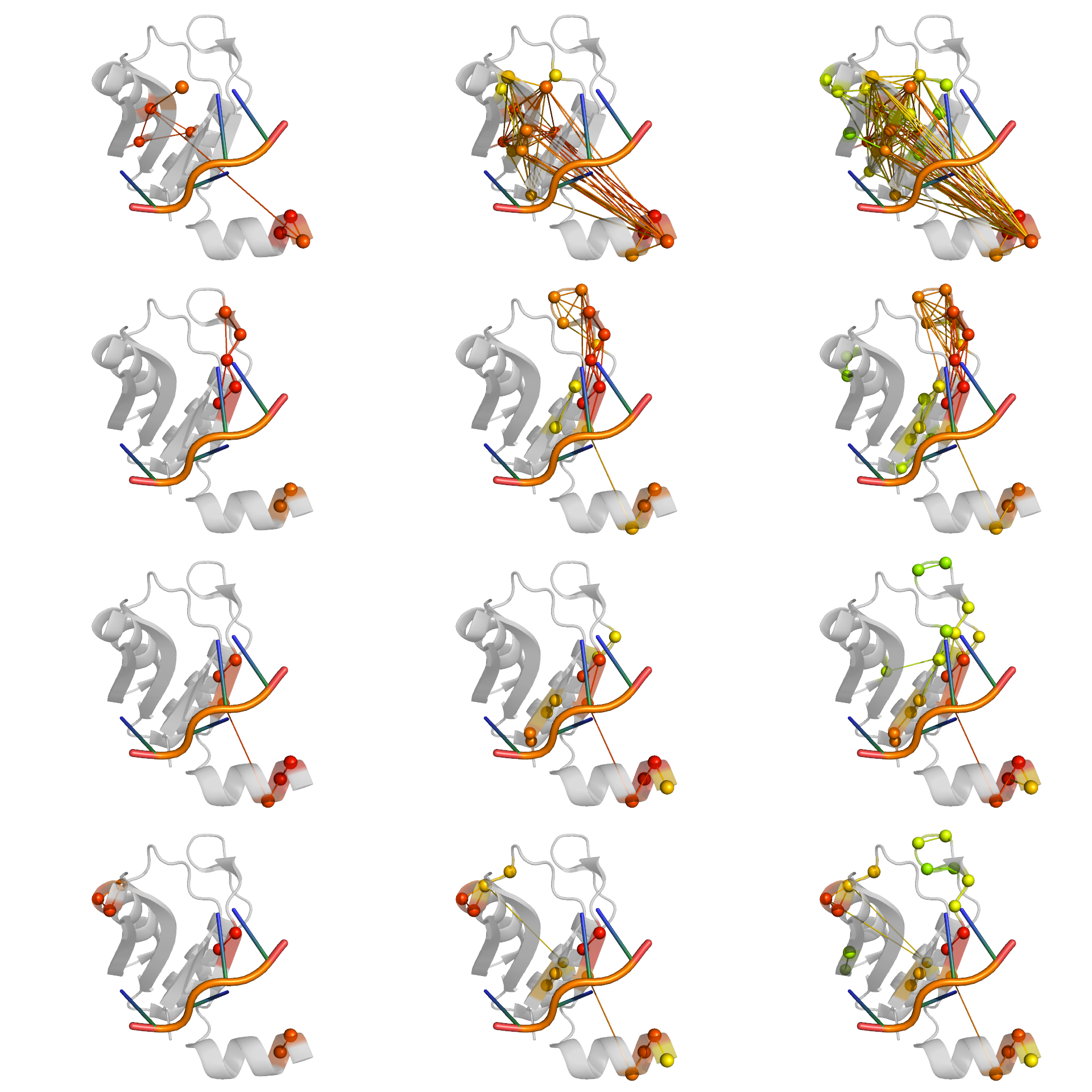


**Supplementary Figure 4. CovET creates a network which connects residues throughout the active site of the RRM domain while other methods create disconnected or localized networks.** For each covariation method residues and their predicted covariation were visualized by adding pairs until the specified coverage (10, 20, or 30%) of residues which map between the query sequence and the structure had been reached. Residues are colored by the percent coverage at which they were added to the structure (0% - warmer colors i.e. red, and 30% - cooler colors i.e. green) and the alpha carbon for each colored residue is shown as a sphere. Predicted covarying pairs are indicated by colored connections between residues where each pair is colored with the same colored as the most recently added (higher % coverage) residue. For all visualizations the PDB 4f02 was used with chain A modeling RRM1 and RRM2 (the query) and chain B the bound mRNA molecule (“atomic” coloring). These structural views show that top ranked CovET pairs form a network throughout the active site of the domain which is highly connected. ET-MIp builds an interconnected network but avoids most of the key residues of the RNA binding sites. EVC and DCA pick up few residues in the active site and their networks are disconnected, including several discrete pairs.

30% Coverage

20% Coverage

10% Coverage

EVC

ET-MIp

CovET

DCA


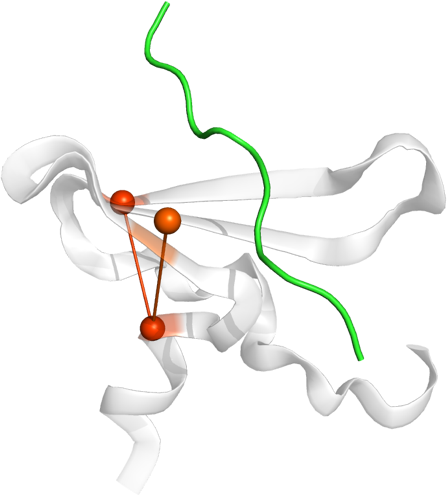

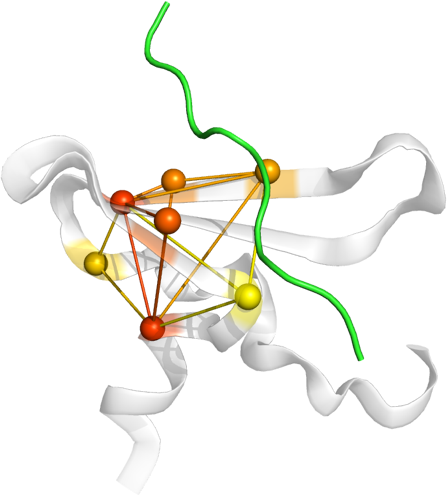

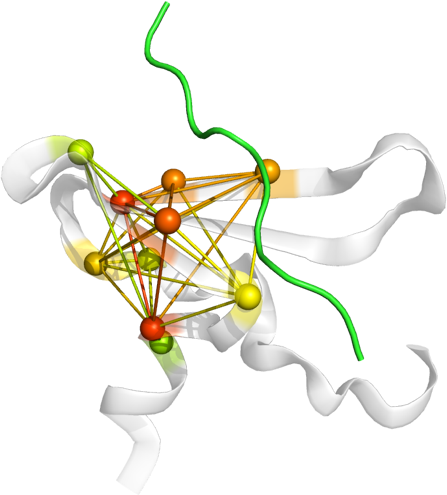

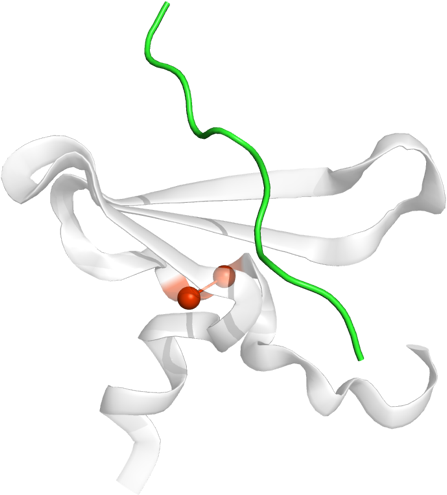

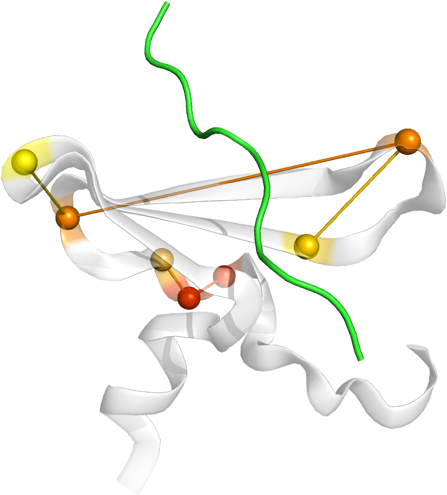

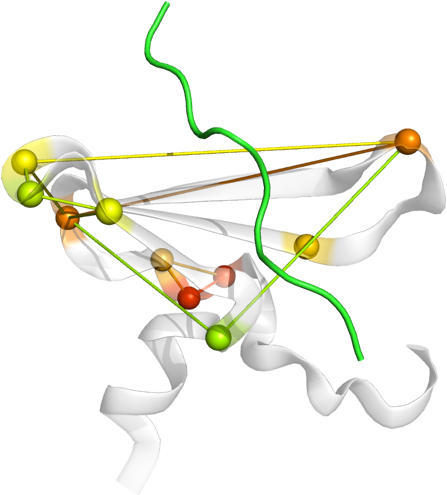

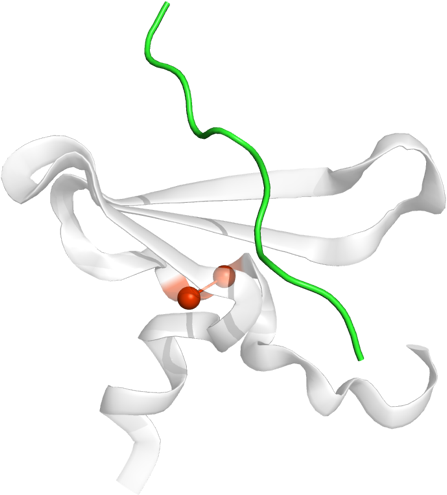

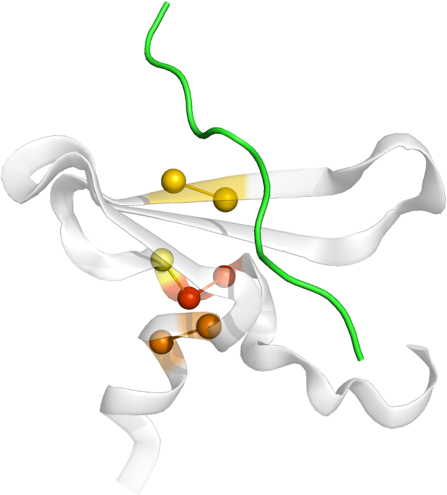

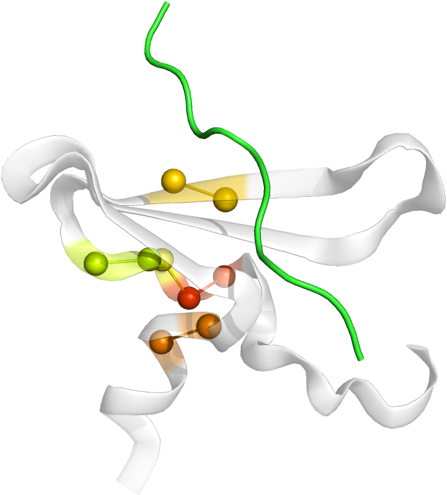

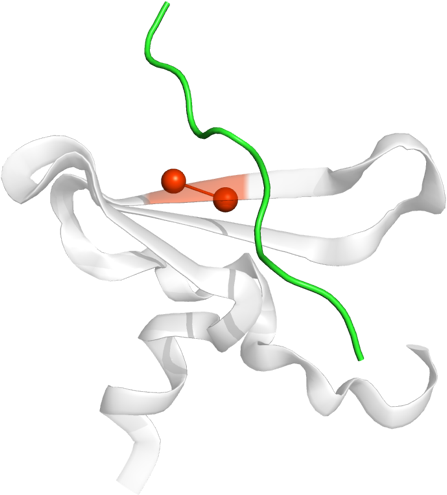

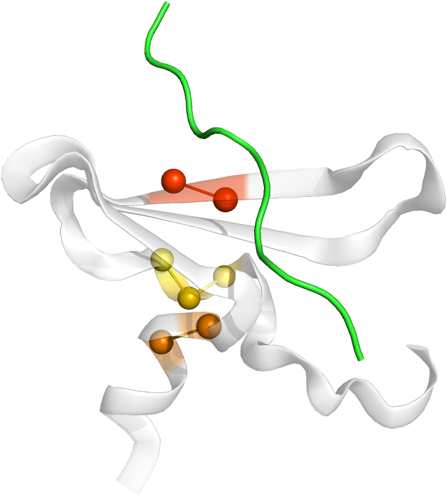

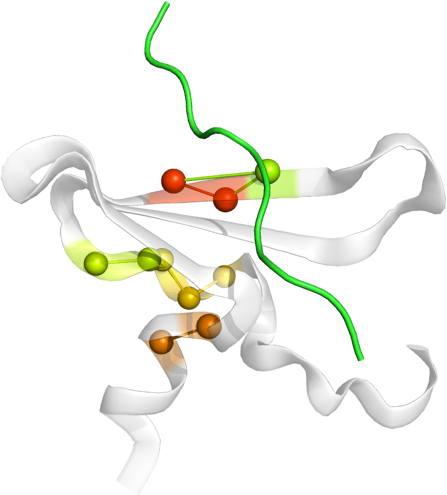


A

B

C

D

**Supplementary Figure 5. CovET creates a network which connects residues throughout the active site of the WW domain while other methods create disconnected or localized networks in the N and C termini.** For each covariation method residues and their predicted covariation were visualized by adding pairs until the specified coverage (2.5, 5, 7,5, 10, 20, or 30%) of residues which map between the query sequence and the structure had been reached. Residues are colored by the percent coverage at which they were added to the structure (0% - warmer colors i.e. red, and 30% - cooler colors i.e. green) and the alpha carbon for each colored residue is shown as a sphere. Predicted covarying pairs are indicated by colored connections between residues where each pair is colored with the same colored as the most recently added (higher % coverage) residue. For all visualizations, the binding peptide shown in green from PDB structure 1jmq (chain P) is used and 4rex (chain A) is used for the WW domain itself. These structural views show that top ranked CovET pairs form a network throughout the active site of the domain which is highly connected. ET-MIp builds an interconnected network but avoids most of the key residues of the binding sites, picking up the turns connecting the β-sheets and residues in the termini instead. Similarly, EVC and DCA pick up few residues in the active site, capturing mostly residues in the terminal region. The networks created by these two methods are also disconnected, consisting of discrete pairs. The number of residues recovered at each threshold varies between methods since there are 35 residues mapped to the PDB, 10% Coverage is defined as three residues. Since the two top CovET pairs share a common residue, both pairs are shown. The top two pairs of all other methods do not share a common residue, so only the top pair is shown.

30%

20%

10%

7.5%

EVC

ET-MIp

CovET

DCA

5%

2.5%


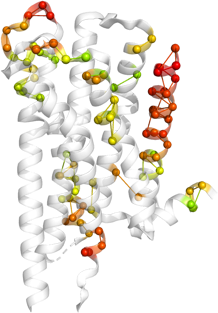

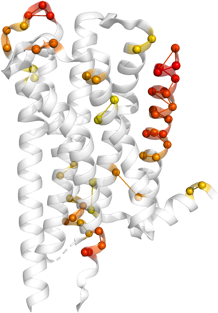

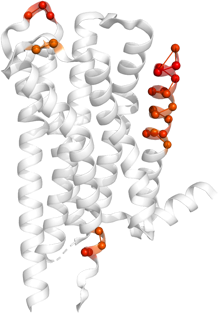

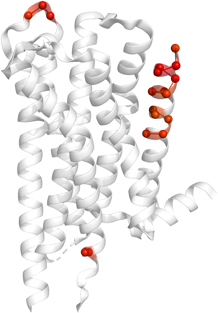

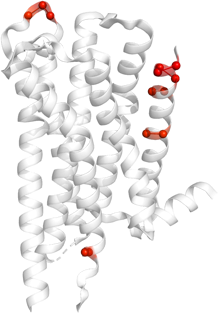

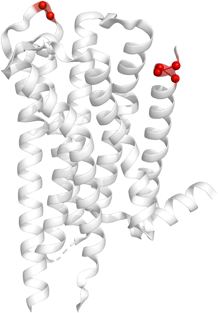

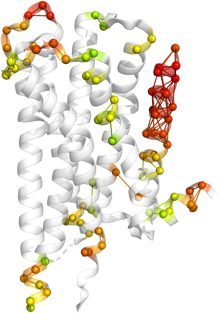

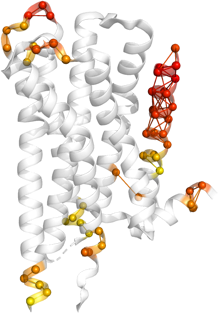

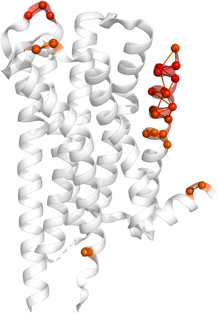

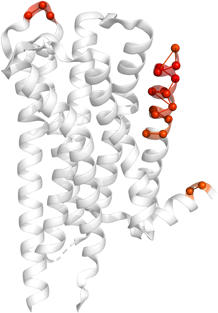

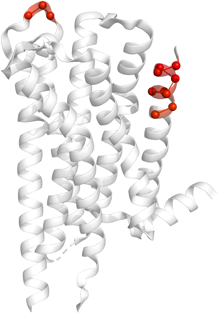

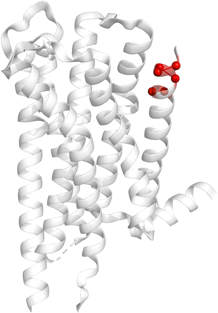

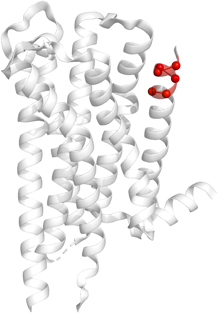

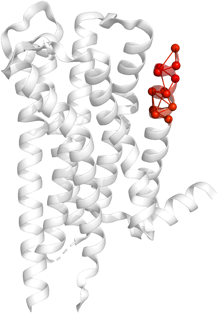

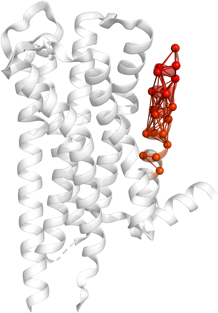

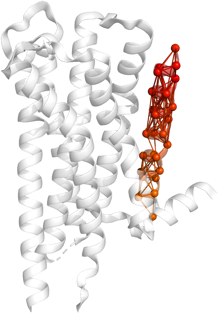

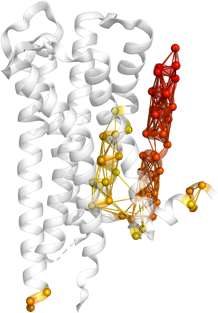

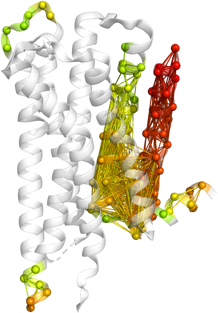

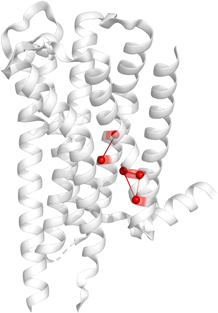

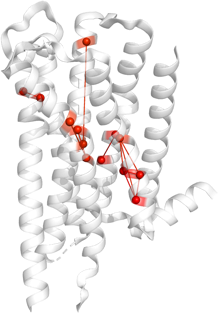

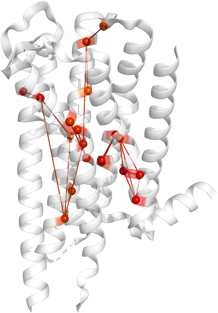

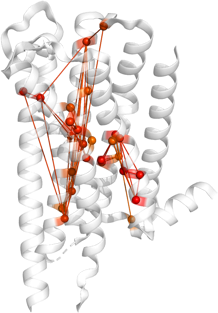

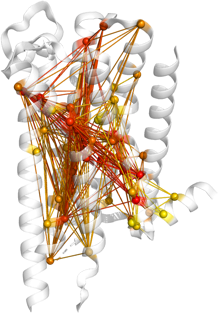

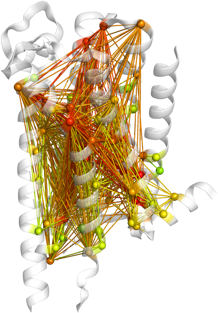


12%

14%

Coverage

16%


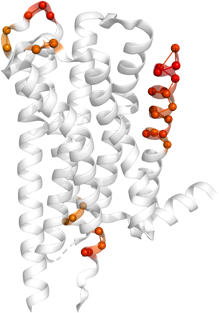

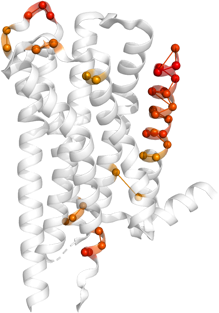

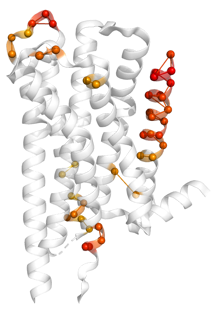

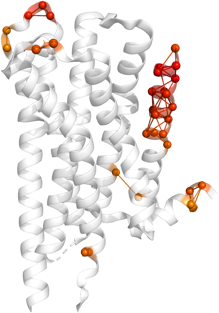

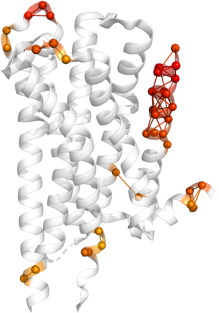

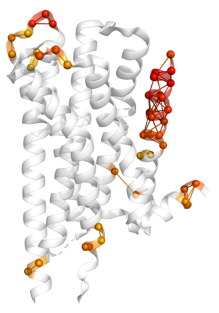

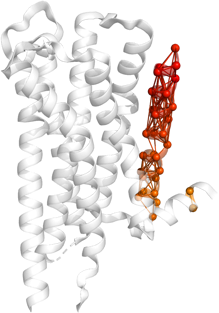

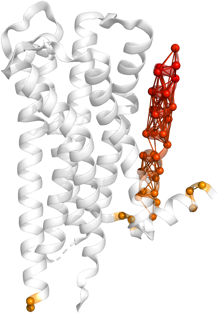

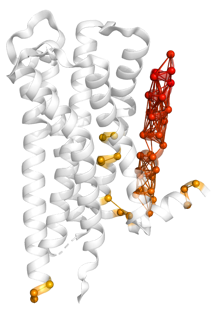

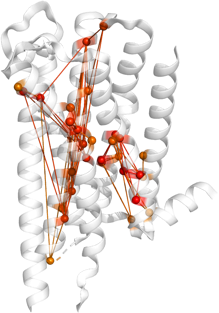

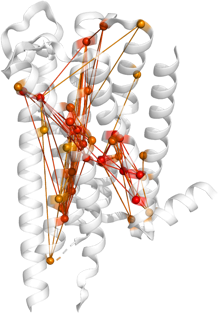

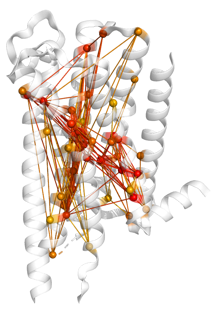


A

B

C

D

**Supplementary Figure 6. CovET creates a network which connects residues throughout the core of the Dopamine D2 Receptor while other methods create disconnected or localized networks.** For each covariation method residues and their predicted covariation were visualized by adding pairs until the specified coverage (2.5, 5, 7,5, 10, 20, or 30%) of residues which map between the query sequence and the structure had been reached. Residues are colored by the percent coverage at which they were added to the structure (0% - warmer colors i.e. red, and 30% - cooler colors i.e. green) and the alpha carbon for each colored residue is shown as a sphere. Predicted covarying pairs are indicated by colored connections between residues where each pair is colored with the same colored as the most recently added (higher % coverage) residue. For all visualizations the PDB 6cm4 was used with chain A modeling the seven transmembrane domain of the D2 Dopamine Receptor (the query). These structural views show that top ranked CovET pairs form a network throughout the core of the receptor which is highly connected. ET-MIp builds an interconnected network but is extremely localized first to transmembrane helix 1 and at coverage cutoff 30% transmembrane helix 2. EVC and DCA are also more localized to transmembrane helix 1 but not to the same degree as ET-MIp, they produce disconnected networks spread out over the transmembrane region with many small groups of residues and isolated pairs.


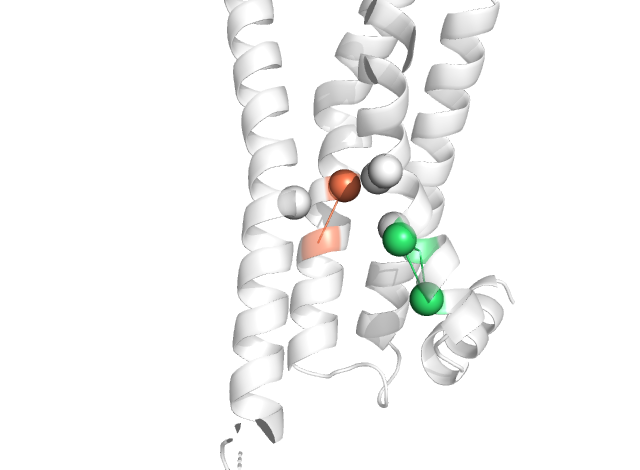


2.5%

Na^+^ Binding Cavity


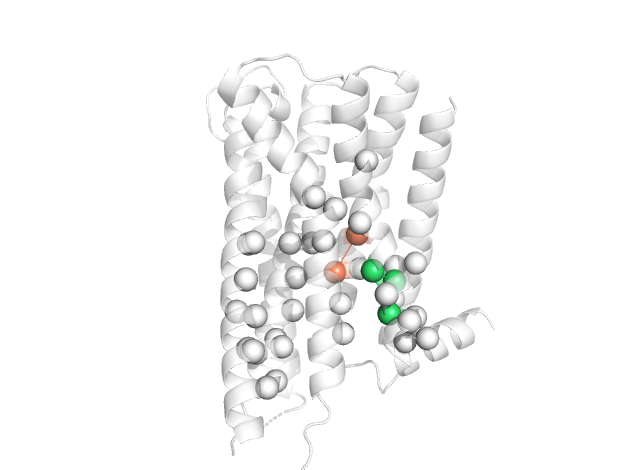


2.5%

State Determinants


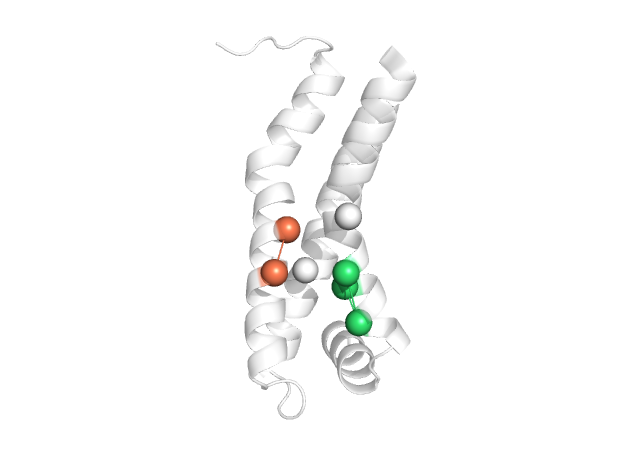


2.5%

Water Channel

5%

CWxP

5%

PIFW


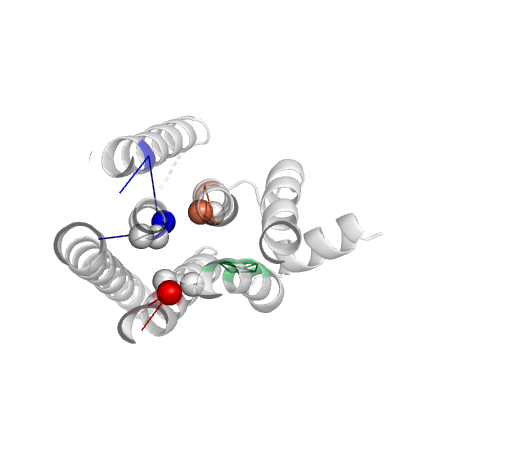


7.5%

HHM

7.5%

Switches


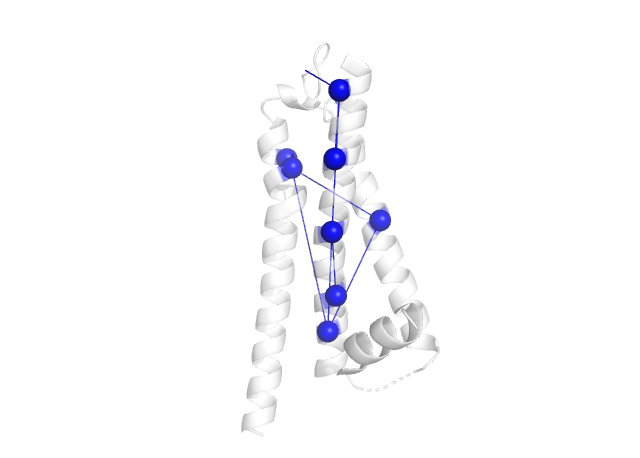


7.5%

TM3


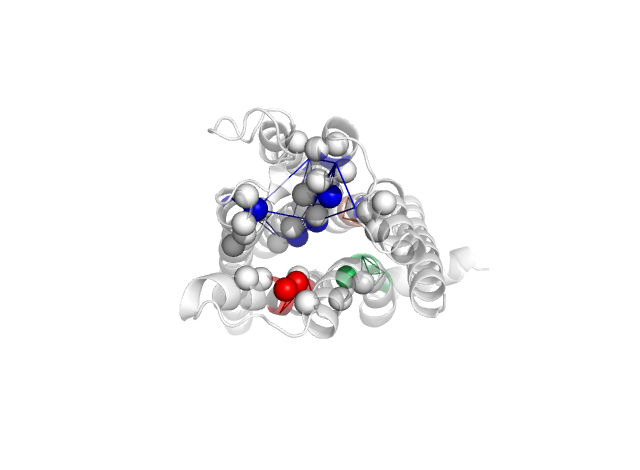


10%

Ligand Binding Site


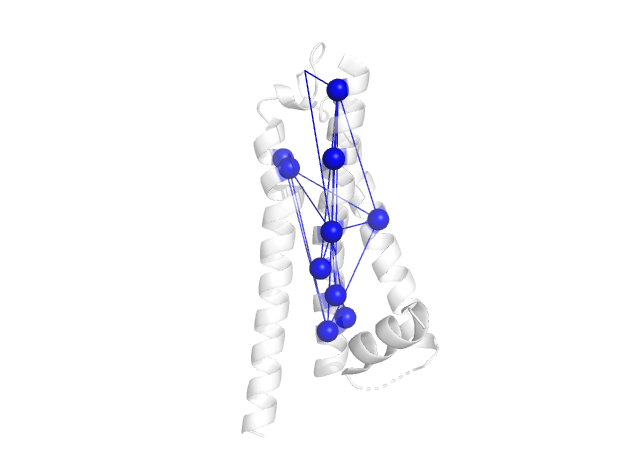


10%

TM3


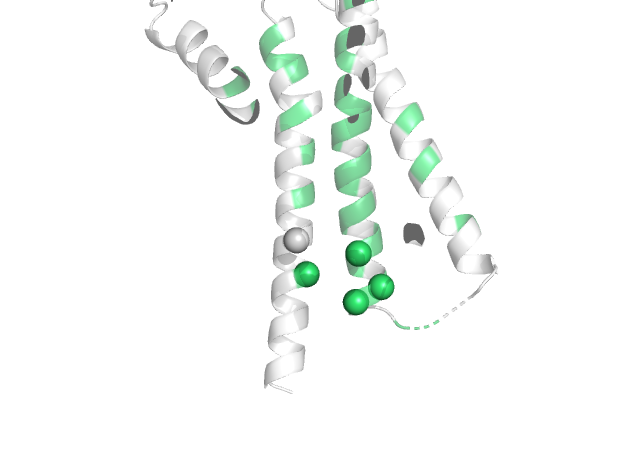


30%

G-Protein Coupling Site


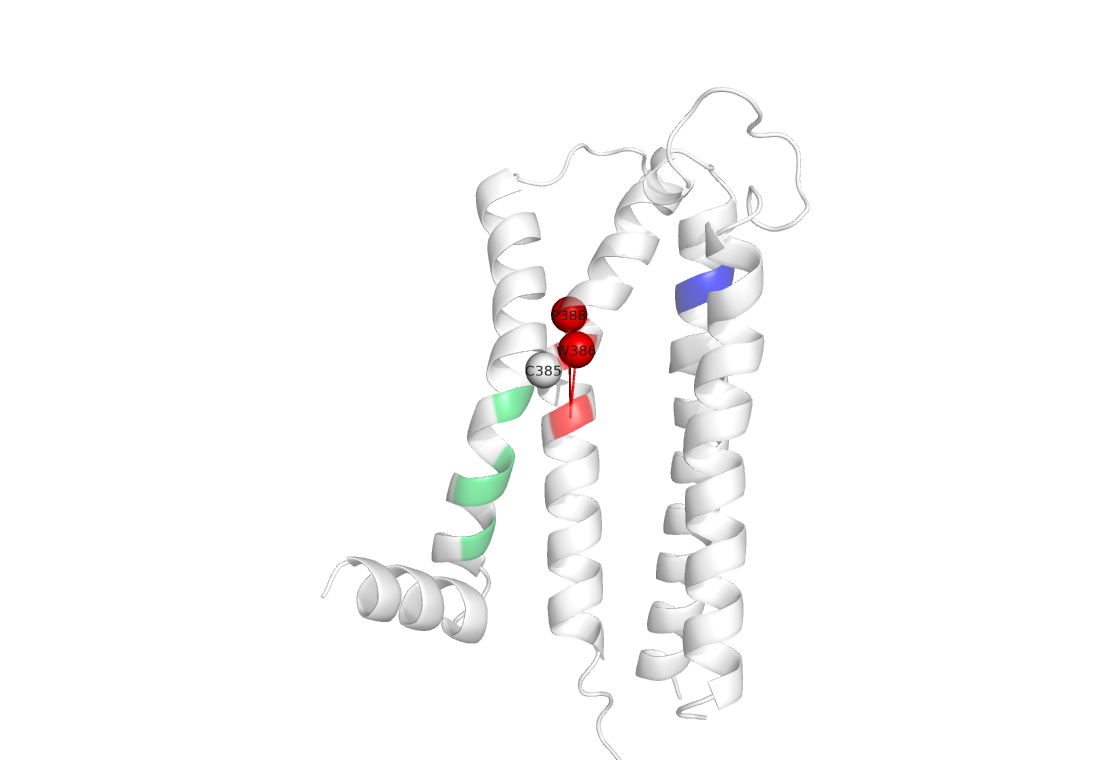

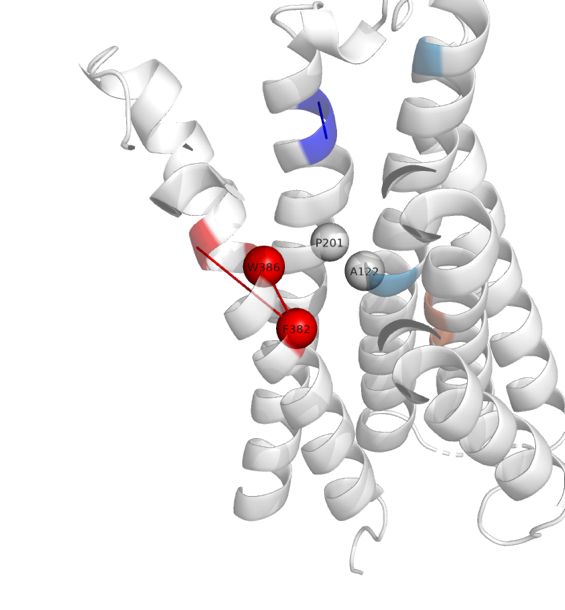

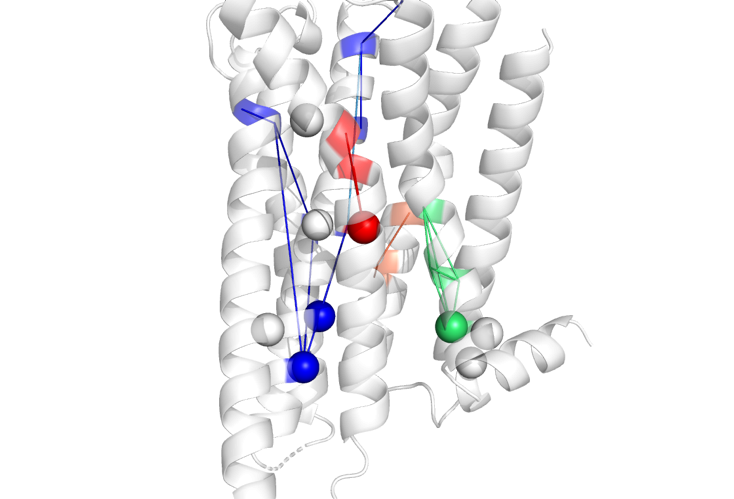


7.5%

Ionic Lock


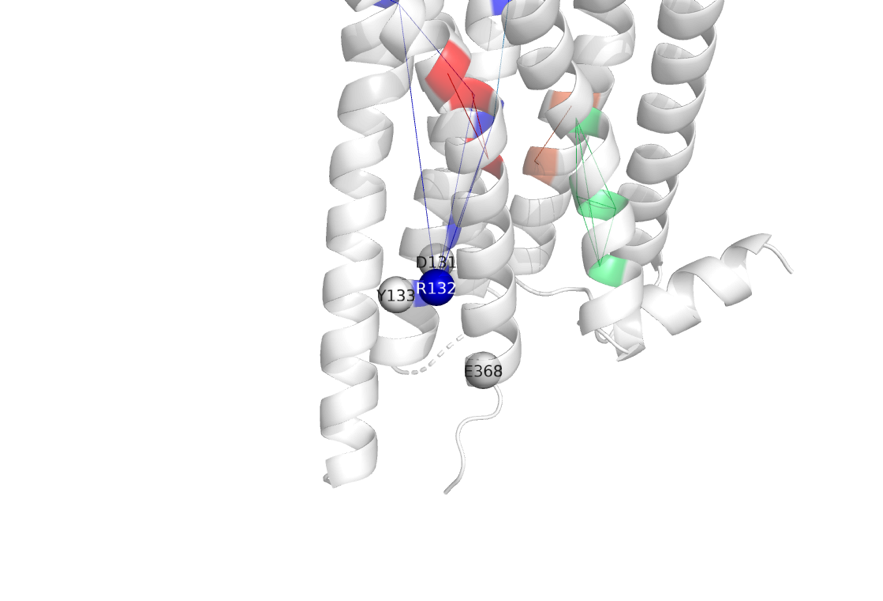


**Supplementary Figure 7. Overlap of CovET discrete clusters with known functional sites in D2R.** CovET identifies discrete clusters for D2R at different coverages. Some of these clusters overlap with known functional sits. Clusters are colored the same as the ones shown in Figure 4. Known functional sites are displayed as spheres.


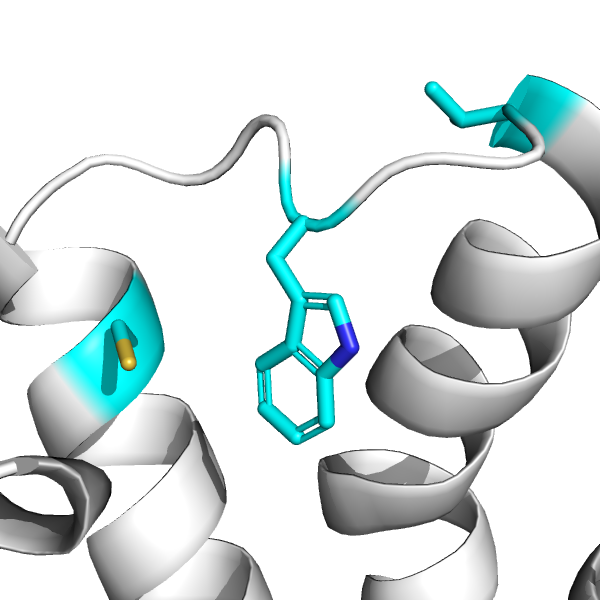

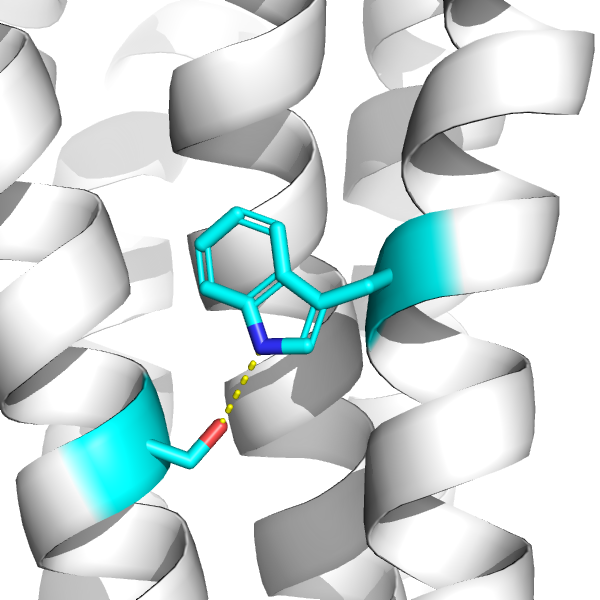


Ser 75

Trp 160

Cys 107

Trp 100

Val 97

A

B

**Supplementary Figure 8. Additional sites identified by CovET in D2R suggest potential targets for further mutagenesis studies.** Some top CovET residues in the D2R are not found in previously recognized functional positions. (A) Val 97, Trp 100 and Cys 107 form a small local cluster near the protein surface. (B) A hydrogen bond is formed between the side chains of Ser 75 and Trp 160. The nonrandom clustering of these sites suggest they would be of interest for further mutational testing.

**Supplementary Tables**

**Supplementary Table 1. Proteins from the Pfam database that are used for assessing structural contacts, and prediction of ligand binding site.** For each protein family that was used in the analysis, the family id, query protein id, query protein sequence, PDB identifier, protein chain, and multiple sequence alignment size are provided.

**Supplementary Table 2. Proteins used to assess functional, and experimental predictive ability of covariation methods.** For each protein, the abbreviation, protein description, original species, query sequence reference (Uniprot ID or PDB), PDB identifier, protein chain, sequence length and multiple sequence alignment size are provided. If a protein domain was used as query, the start and end positions are listed in the “Reference” column.

**Supplementary Table 3. CovET top predictions are enriched for key structural and functional residues in the RRM Domain.** P values measured from the one-sided hypergeometric test were listed.

**Supplementary Tables 4. CovET top predictions are enriched for key structural and functional residues in the WW Domain.** P values measured from the one-sided hypergeometric test were listed.

**Supplementary Table 5. ET-MIp, EVC, and DCA show enrichment for known variable positions in the WW domain.** P values measured from the one-sided hypergeometric test were listed.

**Supplementary Table 6. CovET top predictions are enriched for key structural and functional residues in the Dopamine D2 Receptor.** P values measured from the one-sided hypergeometric test were listed.

**Supplementary Table 7. Top CovET pairs form discrete residue clusters in the Dopamine D2 Receptor.** The residues (PBD: 6CM4) in each discrete cluster are shown. The color for each cluster corresponds to the color in Figure 4.

**Additional References**

Baldassi, C., Zamparo, M., Feinauer, C., Procaccini, A., Zecchina, R., Weigt, M., & Pagnani, A. (2014). Fast and accurate multivariate Gaussian modeling of protein families: Predicting residue contacts and protein-interaction partners. *PLoS ONE*, *9*(3). https://doi.org/10.1371/journal.pone.0092721

Bateman, A. (2019). UniProt: A worldwide hub of protein knowledge. *Nucleic Acids Research*, *47*(D1), D506–D515. https://doi.org/10.1093/nar/gky1049

Camacho, C., Coulouris, G., Avagyan, V., Ma, N., Papadopoulos, J., Bealer, K., & Madden, T. L. (2009). BLAST+: Architecture and applications. *BMC Bioinformatics*, *10*. https://doi.org/10.1186/1471-2105-10-421

Dunn, S. D., Wahl, L. M., & Gloor, G. B. (2008). Mutual information without the influence of phylogeny or entropy dramatically improves residue contact prediction. *Bioinformatics*, *24*(3), 333–340. https://doi.org/10.1093/bioinformatics/btm604

Hopf, T. A., Colwell, L. J., Sheridan, R., Rost, B., Sander, C., & Marks, D. S. (2012). Three-dimensional structures of membrane proteins from genomic sequencing. *Cell*, *149*(7), 1607–1621. https://doi.org/10.1016/j.cell.2012.04.012

Hopf, T. A., Ingraham, J. B., Poelwijk, F. J., Schärfe, C. P. I., Springer, M., Sander, C., & Marks, D. S. (2017). Mutation effects predicted from sequence co-variation. *Nature Biotechnology*, *35*(2), 128–135. https://doi.org/10.1038/nbt.3769

Hopf, T. A., Schärfe, C. P. I., Rodrigues, J. P. G. L. M., Green, A. G., Kohlbacher, O., Sander, C., Bonvin, A. M. J. J., & Marks, D. S. (2014). Sequence co-evolution gives 3D contacts and structures of protein complexes. *ELife*, *3*. https://doi.org/10.7554/eLife.03430

Larkin, M. A., Blackshields, G., Brown, N. P., Chenna, R., Mcgettigan, P. A., McWilliam, H., Valentin, F., Wallace, I. M., Wilm, A., Lopez, R., Thompson, J. D., Gibson, T. J., & Higgins, D. G. (2007). Clustal W and Clustal X version 2.0. *Bioinformatics*, *23*(21), 2947–2948. https://doi.org/10.1093/bioinformatics/btm404

Lichtarge, O., Sowa, M. E., & Philippi, A. (2002). Evolutionary traces of functional surfaces along G protein signaling pathway. In *Methods in Enzymology* (Vol. 344, pp. 536–556). Academic Press Inc. https://doi.org/10.1016/S0076-6879(02)44739-8

Madabushi, S., Yao, H., Marsh, M., Kristensen, D. M., Philippi, A., Sowa, M. E., & Lichtarge, O. (2002). Structural clusters of evolutionary trace residues are statistically significant and common in proteins. *Journal of Molecular Biology*, *316*(1), 139–154. https://doi.org/10.1006/jmbi.2001.5327

Marks, D. S., Colwell, L. J., Sheridan, R., Hopf, T. A., Pagnani, A., Zecchina, R., & Sander, C. (2011). Protein 3D structure computed from evolutionary sequence variation. *PLoS ONE*, *6*(12). https://doi.org/10.1371/journal.pone.0028766

Marks, D. S., Hopf, T. A., & Sander, C. (2012). Protein structure prediction from sequence variation. In *Nature Biotechnology* (Vol. 30, Issue 11, pp. 1072–1080). Nature Publishing Group. https://doi.org/10.1038/nbt.2419

Martin, L. C., Gloor, G. B., Dunn, S. D., & Wahl, L. M. (2005). Using information theory to search for co-evolving residues in proteins. *Bioinformatics (Oxford, England)*, *21*(22), 4116–4124. https://doi.org/10.1093/bioinformatics/bti671

Mihalek, I., Reš, I., & Lichtarge, O. (2004). A Family of Evolution-Entropy Hybrid Methods for Ranking Protein Residues by Importance. *Journal of Molecular Biology*, *336*(5), 1265–1282. https://doi.org/10.1016/j.jmb.2003.12.078

Sung, Y.-M., Wilkins, A. D., Rodriguez, G. J., Wensel, T. G., & Lichtarge, O. (2016). Intramolecular allosteric communication in dopamine D2 receptor revealed by evolutionary amino acid covariation. *Proceedings of the National Academy of Sciences of the United States of America*, *113*(13), 3539–3544. https://doi.org/10.1073/pnas.1516579113
